# EphB2-dependent signaling promotes neuronal excitotoxicity and inflammation in the acute phase of ischemic stroke

**DOI:** 10.1186/s40478-019-0669-7

**Published:** 2019-02-05

**Authors:** Anne-Sophie Ernst, Laura-Inés Böhler, Anna M. Hagenston, Angelika Hoffmann, Sabine Heiland, Carsten Sticht, Martin Bendszus, Markus Hecker, Hilmar Bading, Hugo H. Marti, Thomas Korff, Reiner Kunze

**Affiliations:** 1 Department of Cardiovascular Physiology, Institute of Physiology and Pathophysiology, Heidelberg University, Im Neuenheimer Feld 326, 69120 Heidelberg, Germany; 2 Department of Neurobiology, Interdisciplinary Center for Neurosciences, Heidelberg University, Heidelberg, Germany; 3 Department of Neuroradiology, Heidelberg University Hospital, Heidelberg, Germany; 4 Division of Experimental Radiology, Department of Neuroradiology, Heidelberg University Hospital, Heidelberg, Germany; 5 Center of Medical Research, Medical Faculty Mannheim, Heidelberg University, Heidelberg, Germany

**Keywords:** EphB2, Ephrin-B2, Excitotoxicity, Inflammation, Ischemic stroke, NMDA

## Abstract

Local cerebral hypoperfusion causes ischemic stroke while driving multiple cell-specific responses including inflammation, glutamate-induced neurotoxicity mediated via NMDAR, edema formation and angiogenesis. Despite the relevance of these pathophysiological mechanisms for disease progression and outcome, molecular determinants controlling the onset of these processes are only partially understood. In this context, our study intended to investigate the functional role of EphB2, a receptor tyrosine kinase that is crucial for synapse function and binds to membrane-associated ephrin-B ligands.

Cerebral ischemia was induced in *Ephb2*^*−/−*^ mice by transient middle cerebral artery occlusion followed by different times (6, 12, 24 and 48 h) of reperfusion. Histological, neurofunctional and transcriptome analyses indicated an increase in EphB2 phosphorylation under these conditions and attenuated progression of stroke in *Ephb2*^*−/−*^ mice. Moreover, while infiltration of microglia/macrophages and astrocytes into the peri-infarct region was not altered, expression of the pro-inflammatory mediators MCP-1 and IL-6 was decreased in these mice. In vitro analyses indicated that binding of EphB2 to astrocytic ephrin-B ligands stimulates NF-κB-mediated cytokine expression via the MAPK pathway. Further magnetic resonance imaging of the *Ephb2*^*−/−*^ ischemic brain revealed a lower level of cytotoxic edema formation within 6 h upon onset of reperfusion. On the mechanistic level, absence of neuronal EphB2 decreased the mitochondrial Ca^2+^ load upon specific activation of NMDAR but not during synaptic activity. Furthermore, neuron-specific loss of ephrin-B2 reduced the extent of cerebral tissue damage in the acute phase of ischemic stroke.

Collectively, EphB2 may promote the immediate response to an ischemia-reperfusion event in the central nervous system by (i) pro-inflammatory activation of astrocytes via ephrin-B-dependent signaling and (ii) amplification of NMDA-evoked neuronal excitotoxicity.

**Electronic supplementary material:**

The online version of this article (10.1186/s40478-019-0669-7) contains supplementary material, which is available to authorized users.

## Introduction

Cerebral ischemia is one of the leading causes of death and disability in the Western world while effective therapeutic options such as thrombectomy and thrombolysis are limited to a small number of patients only. Therefore, unraveling pathophysiological mechanisms underlying ischemic stroke is needed to develop novel and adjuvant therapies. Ischemic stroke due to the occlusion of a main cerebral artery leads to a critical shortage of oxygen, glucose and further nutrients in the affected brain area. The latter initiates a complex cascade of detrimental events evolving within different temporal and spatial frames. This ischemic cascade encompasses initial bioenergetic failure and ionic imbalance, followed by excitotoxicity, oxidative stress, dysfunction of the blood-brain barrier (BBB), and inflammatory responses, which altogether lead to the progressive death of neurons, glial and endothelial cells [10].

The bioenergetic failure, rapidly occurring due to the lack of oxygen and glucose, results in breakdown of the normal resting potential of neurons, depolarization, and massive glutamate release. Subsequent excessive stimulation of glutamatergic receptors, most notably extrasynaptic N-methyl-D-aspartate receptors (NMDARs), causes calcium overload and mitochondrial dysfunction, which trigger a range of downstream neurotoxic cascades that impair neuronal function or lead to cell death (glutamate excitotoxicity) [18, 47]. Unchecked influx of Na^+^, K^+^, and Ca^2+^ as a result of ischemia-related energy depletion and glutamate-mediated NMDAR overactivation, in turn, drives Cl^−^ influx via chloride channels, and the resultant increase in intracellular osmolarity drives inflow of water leading to osmotic expansion of the cell (cytotoxic edema) [30]. Cytotoxic edema represents a premorbid cellular process that almost inevitably leads to oncotic or necrotic cell death of mostly astrocytes and neurons [30].

Cytotoxic edema also supplies the driving force for the delayed-onset vasogenic edema that is characterized by extravasation and accumulation of fluid in the cerebral parenchyma due to structural BBB disruption [30, 37, 48]. The BBB is a selective barrier formed by endothelial cells that line cerebral microvessels. Tight junctions (TJs) interconnecting adjacent endothelial cells restrict the movement of molecules and cells across the intact BBB. At the molecular level, TJs consist of specialized transmembrane proteins such as occludin and claudin-5 that are anchored to the actin cytoskeleton via the scaffolding protein Zonula occludens (ZO)-1 [24, 32, 46]. During ischemia-reperfusion (I/R) injury, disruption of regular localization along the cellular borders, increased proteolytic degradation, or reduced expression of TJ proteins occurs causing loss of junctional integrity, increased paracellular BBB permeability, and vasogenic edema formation, which is ultimately responsible for brain swelling in the course of ischemic stroke [24, 46].

As a consequence of neuronal cell death, resident immunocompetent cells of the central nervous system (CNS) such as microglia and astrocytes become rapidly activated. Early pro-inflammatory activation of glial and endothelial cells is accompanied by the release of cytokines, chemokines and reactive oxygen species (ROS) that contribute to the delayed recruitment and transmigration of peripheral immune cells such as neutrophils and monocytes across the disrupted BBB causing secondary brain damage [22].

Taken together, the complex ischemic cascade involves specific spatiotemporal interactions of all parenchymal brain cells. Important determinants orchestrating cellular interactions in this context are the erythropoietin-producing human hepatocellular (Eph) receptors and their corresponding ligands (ephrins). The Eph receptors comprise a large family of receptor tyrosine kinases that not only have important roles in the establishment of neuronal and vascular networks during embryonic development, but also coordinate the homeostasis of the adult CNS and many other adult organs [23, 40, 57]. Their ligands are either attached to the cell surface through a glycosylphosphatidylinositol (GPI) linkage and preferentially bind EphA receptors (ephrin-A) or are transmembrane proteins that preferentially bind EphB receptors (ephrin-B). Interactions are promiscuous within each class, and some Eph receptors can also bind to ephrins of the other class. A distinctive feature of Eph-ephrin complexes is their ability to generate bidirectional signals that affect both the receptor-expressing (“forward” signaling) and ephrin-expressing cells (“reverse” signaling) [23, 40, 57]. There are several lines of evidence suggesting an important role of the EphB receptor/ephrin-B ligand system in stroke pathology. Firstly, in glutamatergic neurons, EphB2 receptors were shown to associate with NMDAR at synaptic sites, and their activation by ephrin-B ligands modulates NMDAR-driven Ca^2+^ influx [50]. Secondly, bidirectional signaling between ephrin-B2 on reactive astrocytes and EphB2 on meningeal fibroblasts is an early important event during development of the glial scar in the adult CNS in response to injury [6]. Thirdly, bidirectional signaling between monocytic EphB2 and endothelial cell ephrin-B1 and -B2 promotes adhesion, pro-inflammatory activation, and transmigration of monocytes, while it lowers the integrity of interendothelial cell junctions and provokes a pro-inflammatory phenotype in endothelial cells [5, 26, 31, 41].

In the light of those findings, our present study aimed to investigate the impact of EphB2 on the outcome of ischemic stroke and the responses of brain parenchymal cells.

## Materials and methods

### Mice

All mouse lines were established on a C57Bl/6 background. We used female and male littermate mice that were age-matched between experimental groups. Mice were between 6 and 10 weeks of age. All animal experiments were approved by the local animal welfare committee (Regierungspräsidium Karlsruhe, Germany, permission number: 35–9185.81/G-112/13), conformed to the Guide for the Care and Use of Laboratory Animals published by the US National Institutes of Health, and were performed in accordance with the recently published Animal Research: Reporting In Vivo Experiments (ARRIVE) guidelines (https://www.nc3rs.org.uk/arrive). All mice were housed at constant room temperature (22 ± 2 °C) and relative humidity (50–55%) on a controlled 12:12 h light-dark cycle, and were provided with standard laboratory chow (LASQCdiet Rod16; LASvendi, Soest, Germany) and water ad libitum. Mice homozygous for a null allele of *Ephb2* (Ephb2^tm1Paw^; *Ephb2*^*−/−*^) [21] and corresponding wild-type (WT) littermates were obtained by mating *Ephb2* haploinsufficient (*Ephb2*^*+/−*^) mice. Depletion of EphB2 protein was confirmed by capillary electrophoresis (Additional file 1: Figure S1a). Neural cell-specific ephrin-B2 deficient mice (*nEfnb2*^*Δ/Δ*^) were generated by crossing animals harboring two floxed alleles (exon 2 flanked by *lox*P sites) of the *Efnb2* gene (B6.E14-TgH(efnb2^flx/flx^)RK; *Efnb2*^*fl/fl*^) [15] with transgenic mice expressing Cre recombinase under control of the promoter and the nervous system-specific enhancer present in the second intron of the rat *nestin* gene (B6.Cg-Tg(Nes-cre)^1Kln^) [52]. Cre-mediated excision of floxed exon 2 in the *Efnb2* gene was successfully verified on the mRNA level using real-time RT-PCR (Additional file 1: Figure S1b). Mice were genotyped using primers (Eurofins Genomics, Ebersberg, Germany) described in Additional file 2: Table S1. All mice were randomly allocated to experimental groups. Operators and investigators were blinded for mouse genotype in all experiments and analyses. Evaluation of all read-out parameters was done independently and in a blinded fashion.

### Experimental stroke model

Mice were used at the age of 7–9 weeks. Female and male mice were anesthetized by a mixture of 2% isoflurane in, 70% N_2_O and remainder O_2_, and were maintained by reducing the isoflurane concentration to 1.0–1.5%. To induce focal cerebral ischemia, a 7–0 silicon rubber-coated nylon monofilament (Doccol Corporation, Redlands, USA) was introduced in the left internal carotid artery and pushed toward the left middle cerebral artery (MCA) as previously described [27]. In subgroups of mice laser-Doppler flowmetry (LDF) was used to confirm successful MCA occlusion (MCAO) as reported previously [27]. The intraluminal suture was left for 60 min. Subsequently, animals were re-anesthetized and the occluding monofilament was withdrawn to allow reperfusion for 6–72 h. For sham surgery, the mice underwent the same procedure without vessel occlusion. The animals were maintained at 37 °C during and after surgery until they were fully recovered from anesthesia. Then, mice were returned to their solitary cages in a heated (30 °C) environment with free access to food and water for 12 h. During the remaining time animals were kept under normal conditions as described above. Additional file 2: Table S2 lists the criteria resulting in exclusion from end-point analysis.

### Behavioral assessment

Motor coordination and balance were assessed by using the Rotarod performance test. Mice were placed individually on the revolving drum. Once they were balanced, the drum was accelerated from 4 to 40 rpm over the course of 300 s, and the time at which the animal dropped off the drum was determined (maximum testing time 300 s). Mice were trained for three consecutive days (three runs each) and were tested directly before MCAO and 24 h after onset of reperfusion. At indicated time points, neurological function was additionally evaluated using the modified Bederson neurological deficit score, according to the following scoring system: 0, no observable deficit; 1, forelimb flexion; 2, decreased resistance to lateral push; 3, unidirectional circling; 4, no movement [4].

### Magnetic resonance imaging (MRI)

MRI was performed on a dedicated small animal scanner with 9.4 Tesla magnetic field strength (BioSpec 94/20 USR, Bruker, Ettlingen, Germany) using a volume coil for RF transmission and a 4-channel phased-array surface receiver coil. An isoflurane evaporator connected to a supply of compressed air was used for anesthesia. Anesthesia was induced at 2% isoflurane and maintained with 1–1.5%. Animals were placed in prone and fixed positions on an animal holder equipped with a headlock and tooth bar to minimize head motion. Body temperature was maintained using a temperature-controlled heating pad. Respiration was monitored externally with an in-house developed program in LabView (National Instruments Corporation, Austin, Texas, USA).

The imaging protocol included T2-weighted imaging (TEeff/TR = 66 ms/2650 ms, RARE factor = 8, slice thickness 0.5 mm, 13 slices, matrix 256 × 256, in plane resolution 78 × 78 μm), diffusion-weighted imaging (TEeff/TR = 20 ms/3400 ms, slice thickness 0.7 mm, one measurement with b = 0 and 30 diffusion sensitized directions with a b-value of 1500 s/mm^2^, field-of-view 12 × 15 mm, matrix 96 × 128, resolution 125 × 117 μm), as well as quantitative T2 measurements (TE increments of 8 ms from 8 ms to 136 ms, TR = 3100 ms, slice thickness 0.5 mm, matrix 172 × 172, resolution 116 × 116 μm). The real signal component from the Multislice Multiple Spin Echo data were fitted after phase correction and SNR optimized multiple coils signal combination on a voxel-by-voxel basis with the monoexponential function S_0_*e ^–(TE/T2)^ using a nonlinear least-squares fit procedure (MATLAB Release 2012b, The MathWorks, Inc., Natick, Massachusetts, United States). S_0_ is the signal at TE = 0. Apparent diffusion coefficient (ADC) values were obtained from diffusion-weighted images by FSL’s (FMRIB [The Oxford Centre for Functional Magnetic Resonance Imaging of the Brain] Software Library) FDT toolbox (https://fsl.fmrib.ox.ac.uk/fsl/fslwiki/FDT). Image analysis was performed with the software Amira (Visage Imaging, Inc., San Diego, USA). The ischemic lesion on ADC and T2 maps in basal ganglia and cortex was determined by manual region growing using threshold-based pre-segmentation.

### Histopathological analysis

Animals were deeply anesthetized and transcardially perfused with PBS (2 ml/min) for 5 min. Brains were removed and embedded into Tissue-Tek (Sakura Finetek, Staufen, Germany). From each brain, 24 coronal sections (10 μm thickness, 0.4 mm distance) were prepared using a Leica CM1520 cryostat (Leica Biosystems, Wetzlar, Germany) at a constant temperature of − 15 °C, and stained with cresyl violet (Merck Millipore, Darmstadt, Germany, #105235) according to the manufacturer’s instructions. Stained brain slices were digitized, and infarct and edema volume was measured using the image analysis software ImageJ (National Institutes of Health, Bethesda, MD, USA) as described previously [29, 43].

Fluoro-Jade C (FJC) staining of cerebral cryosections (10 μm thickness; + 0.62 to − 0.62 mm relative to Bregma) was used to detect neuronal degeneration following the protocol of manufacturer (Merck Millipore, #AG325). FJC staining was recorded using a Zeiss Axiovert 200 M microscope (Carl Zeiss Microscopy, Göttingen, Germany) with a Hamamatsu ORCA flash 4.0 camera (Hamamatsu Photonics, Herrsching am Ammersee, Germany) by applying TissueFAXS scanning software (TissueGnostics, Vienna, Austria). Nuclei were identified by DAPI staining. Cells showing fluorescent signal for FJC were automatically counted by using TissueQuest 4.0 software (TissueGnostics).

### Analysis of cerebrovascular anatomy

Gross anatomical features of the cerebrovascular architecture were determined as described previously [3].

### Evaluation of BBB permeability

Mice were anesthetized 2 h prior to the end of the reperfusion time, and 100 μl of a pre-warmed (37 °C) solution containing 2% Evans Blue (Sigma-Aldrich, Steinheim, Germany) in 0.9% NaCl were injected into the tail vein. Mice were transcardially perfused, brains harvested and separated into entire left and right hemispheres. Each hemisphere was suspended in 500 μl ice-cold 50% trichloroacetic acid and homogenized with a grinding ball at 30 Hz for 2 min (Mixer Mill MM301; Retsch, Haan, Germany). Tissue lysates were then incubated for 2 h at 4 °C and centrifuged at 16,000×g for 15 min at 4 °C. Evans Blue in the supernatant was measured using a spectrophotometer (Synergy HT; BioTek, Bad Friedrichshall, Germany) at 610 nm and quantified according to a standard curve.

### Glial cell cultures

Primary astroglial and microglial cultures were prepared from neonatal WT, *Ephb2*^*−/−*^ and *nEfnb2*^*Δ/Δ*^ mice (P0-P2). The purity of astrocyte and microglia cultures was about 96% and almost 100%, respectively (Additional file 1: Figure S1c). Details on isolation, culture, treatment, and analysis of microglial phagocytosis are provided in the Supplementary Methods (Additional file 3).

### Neuronal cell culture

Primary dissociated cortical cultures were prepared from newborn WT and *Ephb2*^*−/−*^ mice (P0). The relative portion of neurons within the mixed cultures was about 83% (Additional file 1: Figure S1c). Detailed experimental procedures for isolation, culture, and treatment of neuronal cultures as well as analysis of mitochondrial/cytoplasmic Ca^2+^ concentration and mitochondrial membrane potential can be found in the Supplementary Methods (Additional file 3).

### Immunofluorescence staining

Immunofluorescence staining techniques were applied to determine abundance and subcellular localization of certain proteins and were used to identify different cell types in brain tissue sections and cellular monolayers by detection of cell-specific marker proteins. A detailed description is given in the Supplementary Methods (Additional file 3; see also Additional file 2: Table S3).

### Quantitative real-time RT-PCR analysis

Mice were transcardially perfused with PBS, brains harvested, and a 2-mm-thick tissue slice (− 1.0 to − 3.0 mm relative to bregma) was prepared from each brain and separated into the left and right hemispheres. Total RNA from brain tissue samples or cells was isolated using the TRI reagent (Thermo Fisher Scientific, Dreieich, Germany) according to manufacturer’s instructions. For digestion of residual DNA, 10 μg of total RNA was incubated in a 25 μl reaction mix containing 1x DNase-buffer, 40 U RNasin and 1 U DNase (Promega, Mannheim, Germany) for 30 min at 37 °C. Subsequently, cDNA was synthesized using the Access Reverse Transcription PCR Kit (Promega, #A1260) and quantitative real-time PCR for the target sequences was performed in the Rotor-Gene Q (Qiagen, Hilden, Germany) using the QuantiTect SYBR Green PCR Kit (Qiagen). Fluorescence was monitored (excitation at 470 nm and emission at 530 nm) at the end of the annealing phase. Threshold cycle (Ct) was set within the exponential phase of the PCR. Quantification of the PCR product was done by using the ΔΔCt method. Amplification of the 40S ribosomal protein S12 (*Rps12*) cDNA served as an internal standard. Primers were purchased from Eurofins Genomics (for primer sequences, see Additional file 2: Table S4).

### DNA microarray analysis

Mice were transcardially perfused with PBS, brains extracted, and separated into the left and right hemispheres. Total RNA from brain tissue samples was prepared using the TRI reagent (Thermo Fisher Scientific) according to manufacturer’s instructions followed by additional purification utilizing the RNeasy Mini Kit (Qiagen). RNA was tested by capillary electrophoresis on an Agilent 2100 bioanalyzer (Agilent, Waldbronn, Germany) and high quality was confirmed. Gene expression profiling was performed using arrays of mouse MoGene-2_0-st-type from Affymetrix (Santa Clara, USA). Biotinylated antisense cRNA was then prepared according to the Affymetrix standard labeling protocol with the GeneChip® WT Plus Reagent Kit and the GeneChip® Hybridization, Wash and Stain Kit (both from Affymetrix). Afterwards, hybridization on the chip was performed on a GeneChip Hybridization oven 640, then dyed in the GeneChip Fluidics Station 450 and thereafter scanned with a GeneChip Scanner 3000. All equipment used was from Affymetrix (High Wycombe, UK). A Custom CDF Version 20 with ENTREZ-based gene definitions was used to annotate the arrays [7]. The Raw fluorescence intensity values were normalized by applying quantile normalization and RMA background correction. An ANOVA was performed to identify differentially expressed genes using a commercial software package (SAS JMP10 Genomics, version 7) from SAS (SAS Institute, Cary, NC, USA). A false positive rate of a = 0.05 with FDR correction was taken as the level of significance. Gene Set Enrichment Analysis (GSEA) was used to determine whether defined sets of genes exhibited a statistically significant bias in their distribution within a ranked gene list using the software GSEA [49]. Pathways belonging to various cell functions such as cell cycle or apoptosis were obtained from public external databases (KEGG, http://www.genome.jp/kegg). The raw and normalized data are deposited in the Gene Expression Omnibus database (http://www.ncbi.nlm.nih.gov/geo/; accession No. GSE120565).

### Phospho-receptor tyrosine kinase array

Mice were transcardially perfused with PBS, brains removed, and a 3-mm-thick tissue slice (+ 2.50 ± 0.5 to 0.00 ± 0.5 mm relative to bregma) was prepared from each brain and separated into the left and right hemispheres. The relative level of tyrosine phosphorylation of 39 different receptor tyrosine kinases (RTK) was determined in brain tissue samples using the Proteome Profiler Mouse Phospho-RTK Array Kit (R&D Systems, Wiesbaden, Germany, #ARY014) according to manufacturer’s instructions. Briefly, 500 μl lysis buffer was added to each brain tissue slice, and tissue samples were homogenized mechanically as described above. Following incubation on ice for 10 min, tissue homogenates were centrifuged for 5 min at 16,100×g at 4 °C. Protein concentration in the supernatant was then quantified by Bradford assay, and 250 μg protein/sample was processed further following the protocol of manufacturer.

### Capillary electrophoresis

Mice were transcardially perfused with PBS, brains harvested, and a 2-mm-thick tissue slice (+ 3.0 to + 1.0 mm relative to bregma) was prepared from each brain and separated into the left and right hemispheres. Lysis buffer containing 20 mM Tris (pH 7.6), 250 mM NaCl, 1 mM EDTA, 1 mM EGTA, 1% Triton X-100, 0.5% Nonidet P-40, 1 mM DTT, 1 mM PMSF, and 1% protease inhibitor cocktail (all from Sigma-Aldrich) was added to brain tissue samples or cell monolayer. Tissue samples were homogenized mechanically as reported above. Following incubation on ice for 15 min, tissue and cell homogenates were centrifuged for 15 min at 16,100×g at 4 °C. Protein concentration in the supernatant was then quantified by Bradford assay. Analysis of protein expression was performed according to the Wes User Guide using a Wes instrument from ProteinSimple (San Jose, CA, USA). Briefly, protein samples were diluted with 0.1X sample buffer to a final concentration of 0.5 μg/μl, and were mixed with fluorescent 5x Master Mix and incubated at 95 °C for 5 min. The samples were loaded into the Wes microplate along with a biotinylated protein ladder, blocking reagent, primary antibodies against EphB2 (R&D Systems, #AF467; 1:10) and beta-tubulin (Abcam, Cambridge, UK, #ab6046; 1:500), HRP-conjugated anti-rabbit secondary antibody, luminol peroxide, and washing buffer. The plates and capillary cartridges were loaded into the Wes for electrophoresis and chemiluminescence immunodetection using a CCD camera with default settings: electrophoresis, 375 V, 30 min; blocking, 5 min; primary antibody, 30 min; secondary antibody, 30 min; and camera exposure times, 1 s to 32 s. Compass software (ProteinSimple) was used to acquire and analyze the data and to generate gel images and chemiluminescence signal intensity values. Protein expression is calculated as the chemiluminescence intensity area under the curve.

### Enzyme-linked immunosorbent assay (ELISA)

MCP-1 and TNF protein levels in cell supernatants were measured by quantitative ELISA (R&D Systems, Mouse MCP-1 DuoSet ELISA #DY479–05, Mouse TNF-alpha DuoSet ELISA #DY410–05) according to manufacturer’s instructions.

### Statistical analysis

If not indicated otherwise, all results are expressed as means and displayed on scattered dot plots ± standard deviation (SD). Differences between 2 independent experimental groups were analyzed by two-tailed Student’s *t* tests (normally distributed data) or Mann-Whitney *U* rank-sum tests (ordinal and non-normal data). Differences of one parameter among three or more independent experimental groups were analyzed by either one-way ANOVA followed by a Holm-Sidak’s multiple comparisons test (normally distributed data), or by Kruskal-Wallis *H* test with Dunn’s post hoc test (ordinal and non-normal data). Differences of two parameters among two or more independent/correlated experimental groups were analyzed by two-way (Repeated Measures) ANOVA followed by a Holm-Sidak’s multiple comparisons test (normally distributed data). A probability value of *P* < 0.05 was considered statistically significant. Data plotting and statistical analyses were done with Prism 6 (GraphPad Software, La Jolla, CA, USA).

## Results

### EphB2 deficiency reduces brain tissue injury and functional disabilities after ischemic stroke

**Fig. 1:**
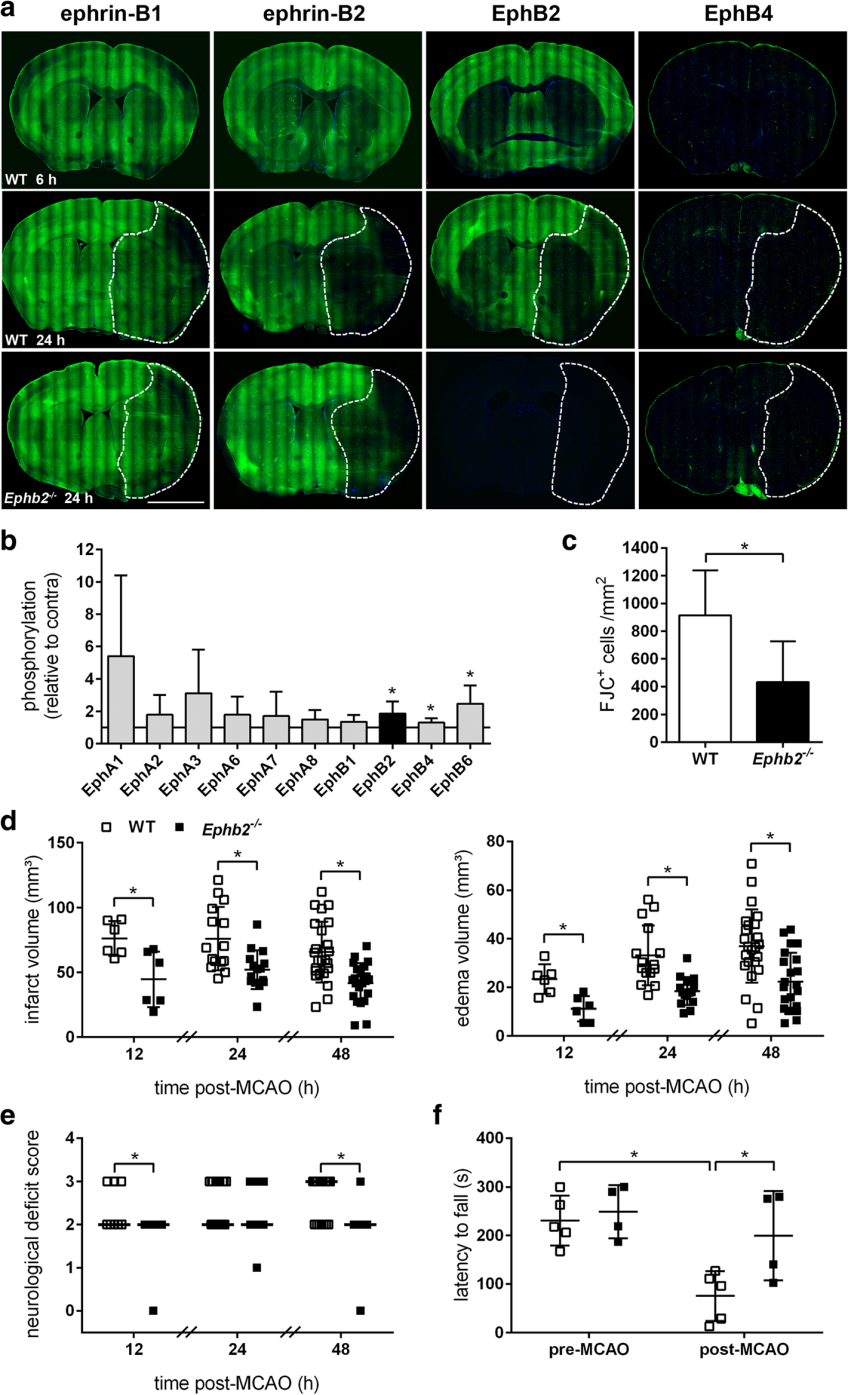
*Ephb2* null mice suffering from acute stroke show reduced brain tissue injury and functional disabilities. **a** WT and *Ephb2*^*−/−*^ mice (*n* = 4/group) were subjected to 60 min MCAO followed by 6 h or 24 h of reperfusion. Immunofluorescent staining was used to determine the spatial distribution of ephrin-B1, ephrin-B2, EphB2 and EphB4 proteins in coronal brain sections. Representative immunofluorescent staining images: ephrin-B/EphB (green) and nuclei (blue). The infarct area according to cresyl violet staining is bordered by a broken white line. Scale bar = 5 mm. **b** WT mice were subjected to 60 min MCAO followed by 6 h of reperfusion. Proteins were isolated from contra-(non-ischemic) and ipsi-(ischemic) lateral brain hemispheres and applied to proteome profiler membranes for detection of phosphorylation levels of Eph receptors (mean ± SD; *n* = 6; Welch’s *t*-test). **(c)** WT and *Ephb2*^*−/−*^ mice underwent 60 min MCAO followed by 12 h of reperfusion. Brain sections were stained with Fluoro-Jade C (FJC), and FJC-positive neurons were counted in the ipsilesional cortex (mean ± SD; *n* = 6; Student’s *t*-test). **d-f** WT and *Ephb2*^*−/−*^ mice were subjected to 60 min MCAO followed by 12, 24 or 48 h of reperfusion as indicated. **d** Brain sections were stained with cresyl violet, and infarct and edema sizes were analyzed using ImageJ. Infarct volume is edema-corrected (single values (scatter blots) and mean ± SD; *n* = 6/6, 15/15, 24/22; Student’s *t*-test). **e** Neurological function was assessed using the Bederson neurological deficit score (single values and median; *n* = 8/11, 17/15, 17/11; Mann-Whitney *U* rank-sum test). **f** Motor coordination of mice subjected to 60 min MCAO followed by 24 h of reperfusion was analyzed by using the Rotarod performance test (single values and mean ± SD; *n* = 5/4; Two-way ANOVA with Holm-Sidak’s multiple comparisons test). * *p* < 0.05

Cerebral ischemia disrupts regular receptor-ligand interactions due to loss of cellular integrity. To investigate whether the spatiotemporal distribution of the ephrin-B/EphB family members is affected under these conditions, we applied immunofluorescence-based analyses. The results indicate a homogenous distribution of ephrin-B1, ephrin-B2 and EphB2 protein across the hemisphere of coronal mouse brain sections, while EphB4 was primarily localized in close proximity to cerebral blood vessels (Fig. 1a). Acute focal ischemic stroke resulted in progressive loss of all ephrin-B/EphB proteins exept EphB4 within both the ipsilesional striatum and adjacent cortical tissue representing the infarct core and ischemic penumbra, respectively (Fig. 1a). Global analysis of receptor tyrosine kinase (RTK) phosphorylation using an antibody array in mice subjected to 60 min of MCAO followed by 6 h reperfusion revealed significantly increased phosphorylation of certain EphB receptor family members including EphB2 within the left ipsilesional cerebral hemisphere (Fig. 1b; phosphorylation status of 39 different murine RTK analyzed post stroke is shown in Additional file 1: Figure S2a) while total EphB2 protein amount did not differ between contra- and ipsilateral brain tissue (Additional file 1: Figure S1a).

To further analyze the role of EphB2, mice homozygous for a null allele of *Ephb2* (*Ephb2*^−/−^) were subjected to I/R injury. While EphB2 was not detectable (Fig. 1a; Additional file 1: Figure S1a), the spatial distribution of ephrin-B1, ephrin-B2, and EphB4 in both ischemic and non-ischemic areas of the mouse brain was not markedly different from WT littermates (Fig. 1a). However, *Ephb2*^−/−^ mice showed markedly reduced neuronal cell death across the infarct lesion during the hyperacute phase of ischemic stroke (Fig. 1c). Global infarct lesion volume and brain swelling due to vasogenic/cytotoxic edema determined 12, 24 and 48 h upon onset of reperfusion were significantly diminished in *Ephb2*^*−/−*^ mice as compared to WT littermates (Fig. 1d). Decreased brain tissue damage in *Ephb2*^−/−^ mice was further associated with reduced Bederson neurological severity scores (Fig. 1e) and improved motor dysfunction (Fig. 1f) during the acute stage of ischemic stroke. Interestingly, when compared to WT littermates, also *Ephb2* haploinsufficient (*Ephb2*^+/−^) mice developed less severe histologic injury and functional impairment after acute cerebral ischemia, albeit not to the same extent as observed in *Ephb2*^*−/−*^ mice (Additional file 1: Figure S2b-d). Collectively, our findings suggest that EphB2 promotes brain tissue from damage during early acute stroke in a gene dosage-dependent manner.

### EphB2 deficiency inhibits the expression of gene sets associated with cell death and inflammation and promotes the expression of gene sets related to synaptic transmission in the CNS after acute ischemic stroke

**Fig. 2:**
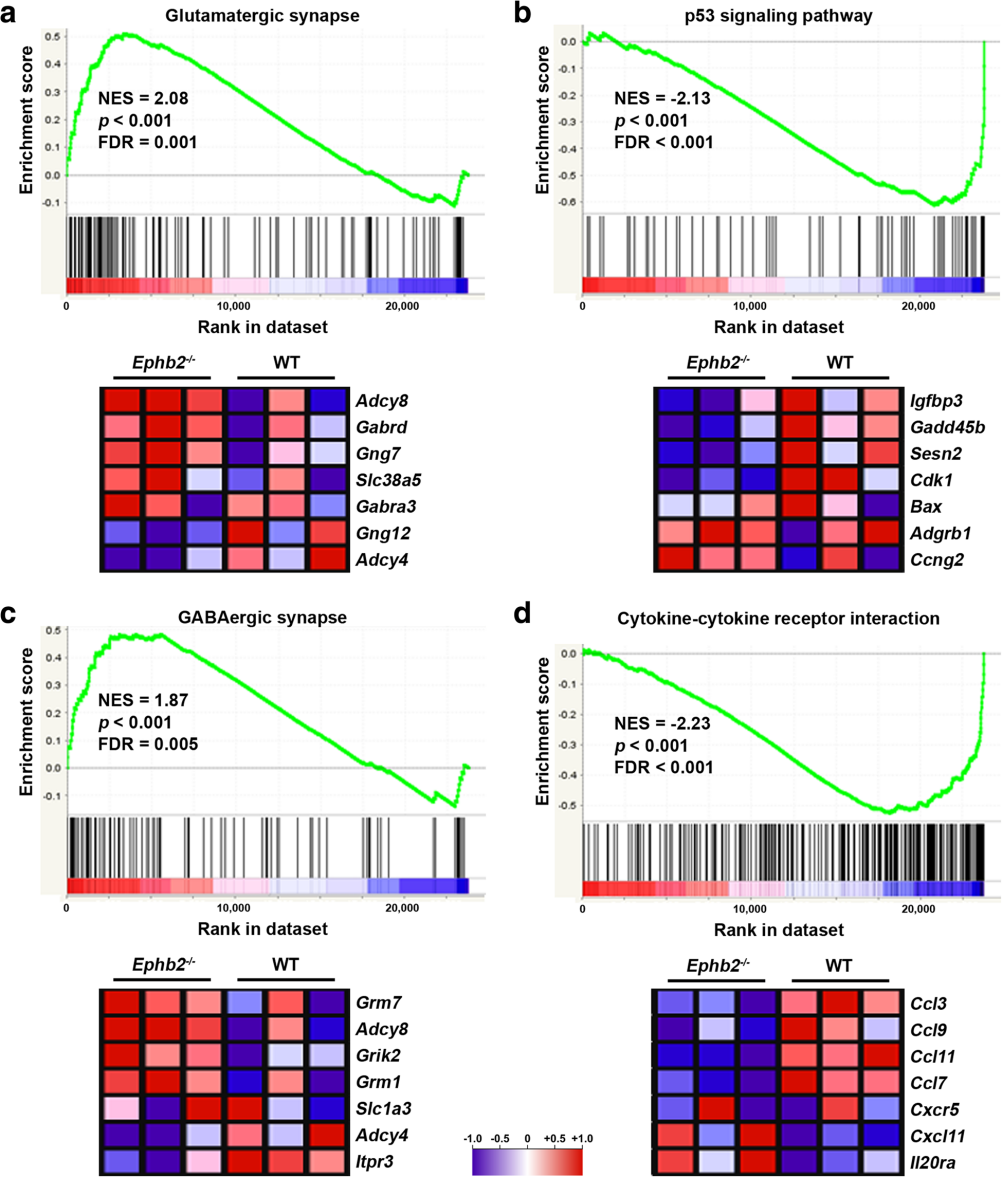
EphB2 deficiency alters gene expression related to cell death, inflammation and synaptic transmission after stroke. WT and *Ephb2*^*−/−*^ mice (*n* = 3/group) were subjected to 60 min MCAO followed by 48 h of reperfusion. Total RNA was isolated from the ipsilateral brain hemisphere and processed for genome array analysis. Exemplary results (enrichment plots) for selected gene sets associated with (**a**, **c**) synaptic transmission, (**b**) cell death and (**d**) inflammation, the corresponding statistical values and excerpt of the corresponding heat maps are shown. NES, normalized enrichment score; *p*, *P*-value (ANOVA); FDR: false discovery rate

We conducted DNA microarray and subsequent gene set enrichment analyses (GSEA) to compare the genome-wide transcriptomic response to acute ischemic stroke in cerebral tissue of *Ephb2*^*−/−*^ and WT mice. Among others, GSEA identified four gene sets linked to cell death that were significantly down-regulated in the ipsilateral brain hemisphere of *Ephb2*^*−/−*^ compared to WT mice (Table 1, Fig. 2; Additional file 2: Table S5). Moreover, twelve gene sets involved in inflammation and immune cell signaling were found to be significantly down-regulated in *Ephb2*^*−/−*^ versus WT littermates (Table 1, Fig. 2; Additional file 2: Table S5). Six gene sets associated with synaptic function were up-regulated in the infarcted brain of *Ephb2*^*−/−*^ in comparison to WT animals (Table 1, Fig. 2; Additional file 2: Table S5).

**Table 1:** KEGG pathway-Based gene set enrichment analyses (GSEA)

Pathway Name	NES	NOM p-val	FDR q-val
Nicotine addiction	2.41	< 0.001	< 0.001
Glutamatergic synapse	2.08	< 0.001	0.001
Morphine addiction	2.03	< 0.001	0.001
Retrograde endocannabinoid signaling	1.97	< 0.001	0.001
GABAergic synapse	1.87	< 0.001	0.005
Axon guidance	1.83	< 0.001	0.007
Circadian entrainment	1.78	< 0.001	0.010
Calcium signaling pathway	1.77	< 0.001	0.011
Cocaine addiction	1.73	0.004	0.015
Salivary secretion	1.56	0.002	0.070
Neuroactive ligand-receptor interaction	1.55	< 0.001	0.072
Parkinson’s disease	1.53	0.002	0.079
Dopaminergic synapse	1.51	0.005	0.082
Platelet activation	− 1.50	0.003	0.048
Central carbon metabolism in cancer	− 1.50	0.020	0.047
Choline metabolism in cancer	−1.50	0.003	0.046
One carbon pool by folate	−1.50	0.042	0.046
Hypertrophic cardiomyopathy (HCM)	−1.51	0.012	0.043
TGF-beta signaling pathway	−1.53	0.014	0.036
Prolactin signaling pathway	−1.54	0.007	0.036
Inflammatory bowel disease (IBD)	−1.55	0.018	0.031
DNA replication	−1.55	0.026	0.031
NOD-like receptor signaling pathway	−1.55	0.011	0.031
Colorectal cancer	−1.55	0.009	0.031
Non-alcoholic fatty liver disease (NAFLD)	−1.56	0.002	0.029
Cell adhesion molecules (CAMs)	−1.58	0.002	0.027
Herpes simplex infection	−1.60	0.002	0.022
Viral myocarditis	−1.61	0.002	0.021
Melanoma	−1.61	0.007	0.021
Bladder cancer	−1.62	0.011	0.020
Galactose metabolism	−1.63	0.011	0.019
Adipocytokine signaling pathway	−1.63	0.002	0.018
Mineral absorption	−1.63	0.008	0.018
Acute myeloid leukemia	−1.64	0.011	0.017
Sphingolipid signaling pathway	−1.65	< 0.001	0.015
MAPK signaling pathway	−1.67	< 0.001	0.013
Pathways in cancer	−1.67	< 0.001	0.013
Hepatitis C	−1.68	< 0.001	0.012
Biosynthesis of amino acids	−1.69	< 0.001	0.011
Protein digestion and absorption	−1.72	< 0.001	0.008
Estrogen signaling pathway	−1.72	< 0.001	0.008
Fc epsilon RI signaling pathway	−1.74	< 0.001	0.007
Transcriptional misregulation in cancer	−1.74	< 0.001	0.007
Natural killer cell mediated cytotoxicity	−1.74	< 0.001	0.007
Glioma	−1.74	< 0.001	0.007
Regulation of actin cytoskeleton	−1.74	< 0.001	0.007
Bacterial invasion of epithelial cells	−1.75	0.002	0.007
Cell cycle	−1.76	< 0.001	0.006
Chemokine signaling pathway	−1.76	< 0.001	0.006
Arginine and proline metabolism	−1.77	< 0.001	0.005
Measles	−1.77	< 0.001	0.006
Pancreatic cancer	−1.78	< 0.001	0.005
Fc gamma R-mediated phagocytosis	−1.79	< 0.001	0.005
HTLV-I infection	−1.79	< 0.001	0.004
FoxO signaling pathway	−1.80	< 0.001	0.004
Chronic myeloid leukemia	−1.82	< 0.001	0.003
Prostate cancer	−1.84	< 0.001	0.003
MicroRNAs in cancer	−1.84	< 0.001	0.003
Hepatitis B	−1.85	< 0.001	0.002
Salmonella infection	−1.85	0.002	0.002
Protein processing in endoplasmic reticulum	−1.87	< 0.001	0.002
VEGF signaling pathway	−1.87	< 0.001	0.002
Epstein-Barr virus infection	−1.87	< 0.001	0.002
Influenza A	−1.88	< 0.001	0.002
Spliceosome	−1.92	< 0.001	0.001
Phagosome	−1.93	< 0.001	0.001
Tuberculosis	−1.93	< 0.001	0.001
Chagas disease (American trypanosomiasis)	−1.94	< 0.001	0.001
Hematopoietic cell lineage	−1.96	< 0.001	0.001
Toll-like receptor signaling pathway	−1.97	< 0.001	0.001
Jak-STAT signaling pathway	−1.97	< 0.001	0.001
PI3K-Akt signaling pathway	−1.97	< 0.001	0.001
Proteasome	−1.98	< 0.001	0.001
Apoptosis	−2.00	< 0.001	0.001
Rheumatoid arthritis	−2.00	< 0.001	0.001
Amoebiasis	−2.00	< 0.001	0.001
Antigen processing and presentation	−2.00	< 0.001	0.001
Pertussis	−2.05	< 0.001	< 0.001
Malaria	−2.06	< 0.001	< 0.001
*Staphylococcus aureus* infection	−2.06	< 0.001	< 0.001
Legionellosis	−2.06	< 0.001	< 0.001
Osteoclast differentiation	−2.07	< 0.001	< 0.001
B cell receptor signaling pathway	−2.07	< 0.001	< 0.001
HIF-1 signaling pathway	−2.08	< 0.001	< 0.001
Leishmaniasis	−2.11	< 0.001	< 0.001
Proteoglycans in cancer	−2.12	< 0.001	< 0.001
p53 signaling pathway	−2.13	< 0.001	< 0.001
Leukocyte transendothelial migration	−2.15	< 0.001	< 0.001
Complement and coagulation cascades	−2.16	< 0.001	< 0.001
TNF signaling pathway	−2.17	< 0.001	< 0.001
NF-kappa B signaling pathway	−2.20	< 0.001	< 0.001
Toxoplasmosis	−2.22	< 0.001	< 0.001
Cytokine-cytokine receptor interaction	−2.23	< 0.001	< 0.001
Small cell lung cancer	−2.27	< 0.001	< 0.001
Focal adhesion	−2.29	< 0.001	< 0.001
ECM-receptor interaction	−2.46	< 0.001	< 0.001

*Ephb2*^*−/−*^ and WT mice (*n* = 3) were subjected to 60 min MCAO followed by 48 h reperfusion. Total RNA was extracted from ipsilesional brain tissue and processed for DNA microarray analysis (GeneChip Mouse Gene 2.0 ST; Affymetrix, Santa Clara, CA, USA). The table lists all significantly UP and DOWN-regulated gene sets comparing *Ephb2*^*−/−*^ versus WT matching the following criteria for selection: ≥1.5- or ≤ 1.5-fold regulation (log_2_-fold), p < 0.05 (ANOVA), FDR < 0.1. NES: normalized enrichment score; FDR: false discovery rate

Overall, these data suggest that EphB2-dependent signaling regulates processes determining the extent of infarction during acute ischemic stroke such as (1) cellular viability, (2) post-ischemic inflammation, and (3) synaptic function.

### Lack of EphB2 ameliorates brain edema following acute ischemic stroke by diminishing the formation of cytotoxic edema

In order to unravel cellular/molecular mechanisms underlying the aforementioned findings in *Ephb2*-deficient mice after acute ischemic stroke, we first investigated a possible vascular mechanism, as a significant proportion of double-mutant mice deficient in both EphB2 and EphB3 receptor exhibit defects in the remodeling of the embryonic vascular system [1]. Perfusion of the arterial system through the left ventricle of the heart using colored particles that cannot pass the capillary system showed a similar gross anatomy of the circle of Willis at the base of the brain from *Ephb2*^*−/−*^ mice versus WT littermates (Additional file 1: Figure S3a). The average number of surface arterial branches that originate from the proximal MCA was also similar between *Ephb2*^*−/−*^ and WT animals (Additional file 1: Figure S3a). Cerebral perfusion monitoring revealed that proximal MCAO resulted in an average reduction of regional cerebral blood flow (rCBF) by 85 ± 6% in WT and 83 ± 7% in *Ephb2*^*−/−*^ mice (Additional file 1: Figure S3b). Moreover, neither the density of cerebral microvessels nor the pericyte coverage of the microvasculature was significantly affected due to ablation of the *Ephb2* gene (Additional file 1: Figure S3c, d). These findings confirm successful arterial occlusion in both genotypes, and exclude the possibility that lack of EphB2 increases the number or diameter of pial collaterals and/or leptomeningeal arteriole–arteriole anastomoses interconnecting distal branches of the MCA, anterior cerebral artery (ACA), and posterior cerebral artery (PCA) trees, which could partially compensate for disturbed blood flow after arterial occlusion [33].

**Fig. 3:**
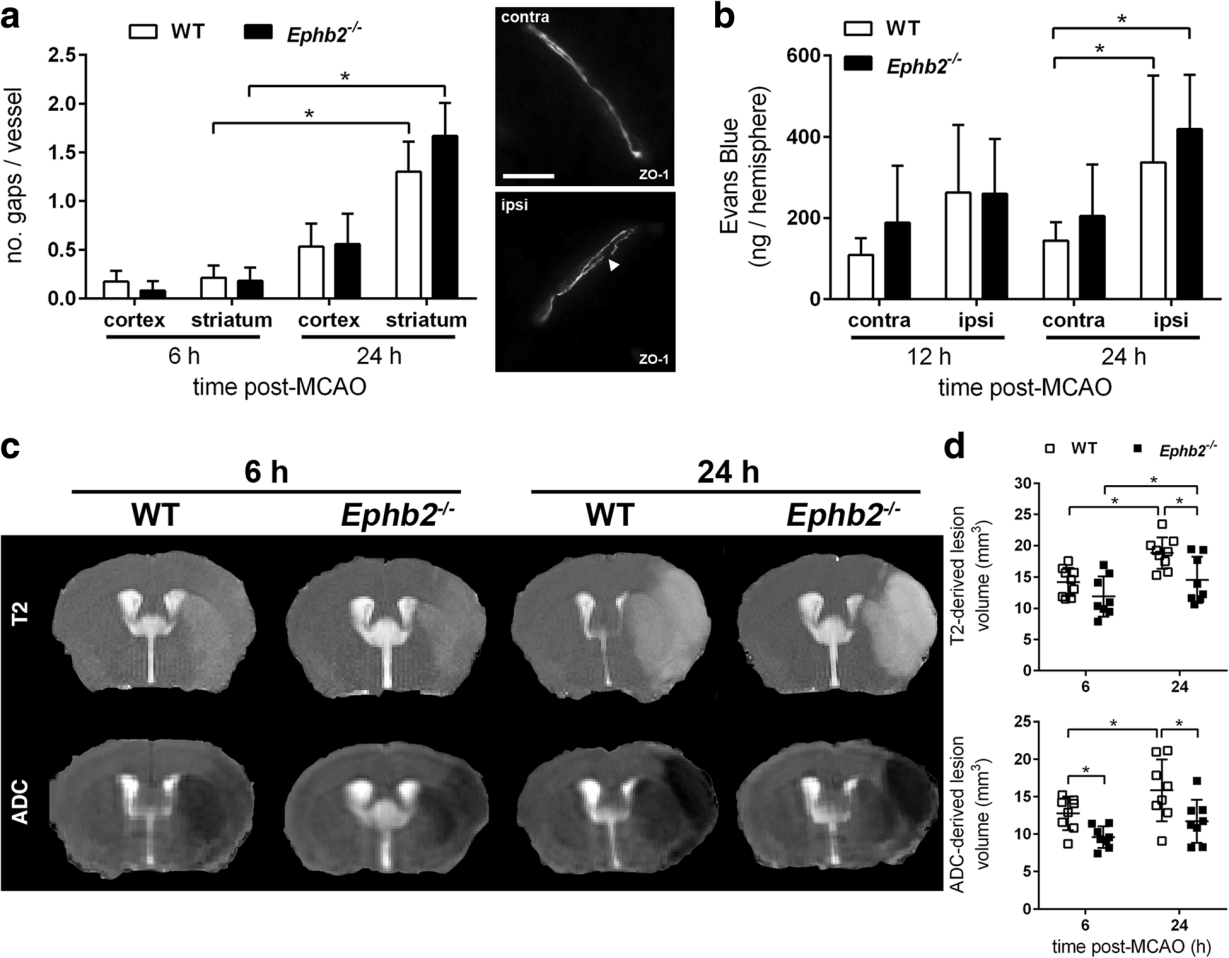
Lack of EphB2 ameliorates brain edema following acute stroke by diminishing formation of cytotoxic edema. **a** WT and *Ephb2*^*−/−*^ mice underwent 60 min MCAO followed by 6 or 24 h of reperfusion. Brain sections were subjected to immunofluorescent staining of CD31 and ZO-1 to visualize interendothelial tight junctions. For each mouse disruptions (gaps; denoted by white arrowhead) of the regular ZO-1 localization pattern were counted in 10–30 randomly chosen microvessels localized within the ipsilesional striatum (infarct core) and cortex (periinfarct), respectively (mean ± SD; *n* = 4/4, *n* = 4/4; Two-way ANOVA with Holm-Sidak’s multiple comparisons test). Scale bar = 10 μm. **b** WT and *Ephb2*^*−/−*^ mice were subjected to 60 min MCAO followed by 12 or 24 h of reperfusion. Evans Blue was applied by tail vein injection 2 h before end of the experiment. After transcardial perfusion the amount of Evans Blue was quantified within contra- and ipsilateral brain hemisphere using absorption spectroscopy (mean ± SD; *n* = 3/4, *n* = 9/8; Two-way ANOVA with Holm-Sidak’s multiple comparisons test). **c**, **d** WT and *Ephb2*^*−/−*^ mice were subjected to 60 min MCAO followed by 24 h of reperfusion. High-field (9.4 T) in vivo MRI was applied to monitor spatiotemporal development of vasogenic (T2-weighted imaging) and cytotoxic (ADC maps derived from diffusion-weighted imaging) edema post-stroke. Representative brain T2- and diffusion-weighted images of WT and *Ephb2*^*−/−*^ mice in coronal planes are depicted in (**c**). **d** shows quantification of the ischemic lesion volume on T2 and ADC maps in basal ganglia (single values and mean ± SD; *n* = 9/8; Two-way ANOVA with Holm-Sidak’s multiple comparisons test). * *p* < 0.05

Our morphovolumetric analyses shown in Fig. 1e revealed reduced brain swelling in *Ephb2*^*−/−*^ mice suffering from acute ischemic stroke. To address a possible role of BBB damage, we analyzed endothelial TJ structures. Our histological analyses disclosed a rearrangement of ZO-1 resulting in progressive gap formation at the endothelial cell membrane of blood vessels located in the ipsilesional cortex and striatum, which, however, was fairly comparable between *Ephb2*^*−/−*^ and WT mice (Fig. 3a). In line with the progressive structural damage of the BBB after restoration of cerebral blood flow, the amount of Evans blue (EB), an intravenously administered intravital dye that binds to plasma albumin, extravasated into the brain parenchyma was markedly increased in ipsilesional as compared to contralateral brain tissue (Fig. 3b). The post-ischemic BBB leakage of EB upon 12 and 24 h of reperfusion was not significantly different between *Ephb2*^*−/−*^ and WT animals (Fig. 3b), suggesting that lack of EphB2 has no relevant direct influence on the BBB hyperpermeability during acute ischemic stroke. In addition to these post-mortem examinations, we utilized multimodal, high-field (9.4 T) in vivo MRI for longitudinal evaluation to assess both vasogenic and cytotoxic edema formation in the brain during the acute phase of stroke using T2 relaxometry and diffusion-weighted imaging, respectively. Apparent diffusion coefficient (ADC) infarct volume (as determined by diffusion-weighted imaging) in the basal ganglia of *Ephb2*^*−/−*^ mice was significantly lower at 6 h after reperfusion compared to WT mice while no volume differences were seen on T2 maps between both groups, suggesting that less cytotoxic edema evolves in *Ephb2*^*−/−*^ mice during early time points after reperfusion (Fig. 3c, d). From 6 to 24 h after reperfusion the ADC and T2 infarct volume increased in both groups but to a significantly lesser degree in *Ephb2*^*−/−*^ mice (Fig. 3c, d).

Altogether, our observations suggest that lack of EphB2 has no effect on basal or infarct-triggered loss of BBB function but may indirectly attenuate stroke-associated brain swelling by reducing early cytotoxic edema, which in turn limits vasogenic edema formation.

### EphB2 deficiency mitigates brain inflammation in the acute stage following ischemic stroke

**Fig. 4:**
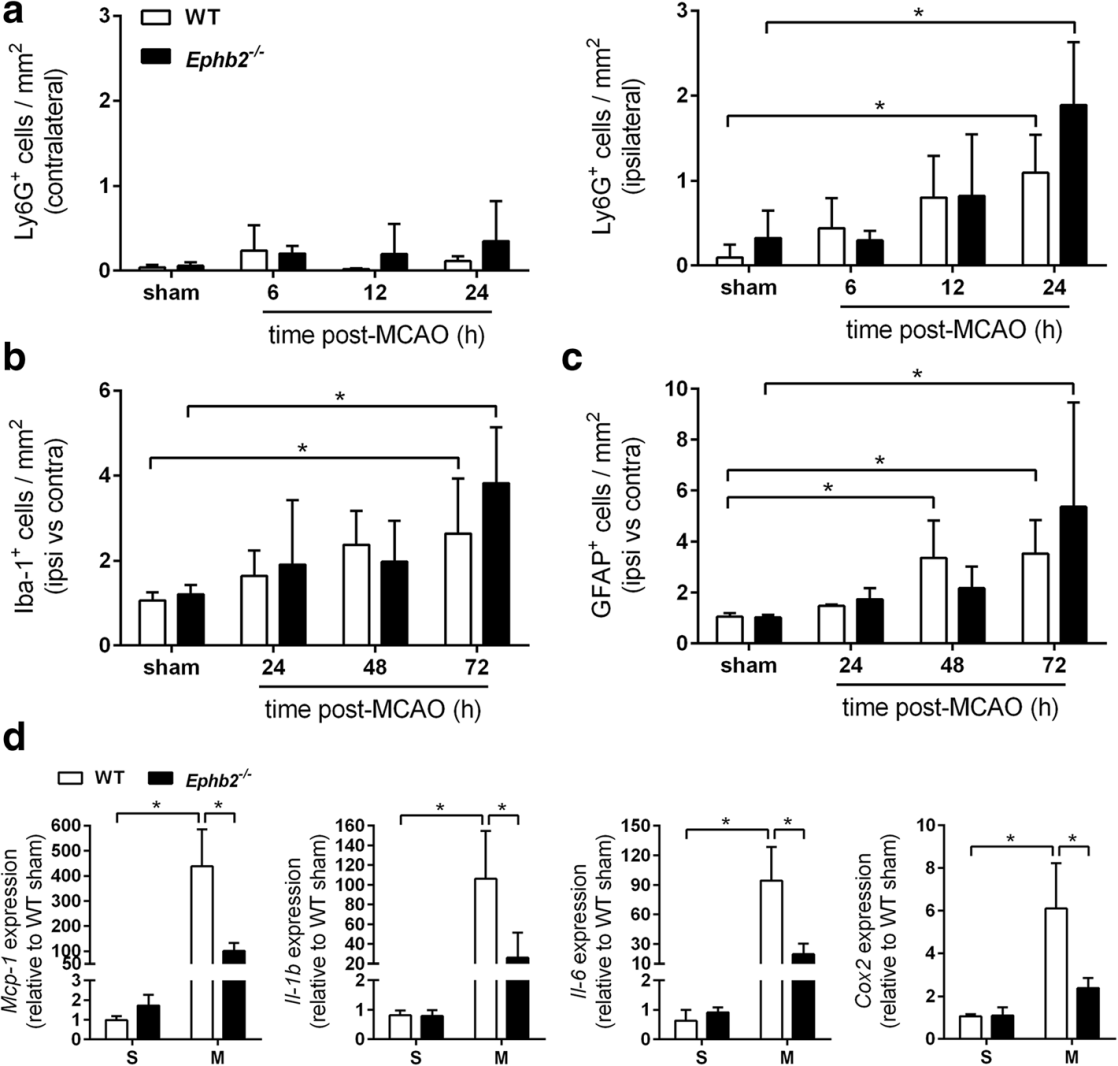
EphB2 deficiency mitigates brain inflammation in the acute stage after ischemic stroke. **a-c** WT and *Ephb2*^*−/−*^ mice underwent 60 min MCAO followed by either 6, 12, 24, 48 or 72 h of reperfusion or were subjected to sham surgery. Immunofluorescent staining of Ly6G, Iba-1 and GFAP was applied to determine the number of (**a**) infiltrating neutrophils within the contra- and ipsilateral brain hemisphere (mean ± SD; *n* = 4/4; Two-way ANOVA with Holm-Sidak’s multiple comparisons test), (**b**) microglia/macrophages and (**c**) astrocytes along the infarct border zone (mean ± SD; *n* = 4/4; Two-way ANOVA with Holm-Sidak’s multiple comparisons test). **d** RNA was extracted from ipsilesional brain tissue and corresponding tissue of sham operated mice. Expression of pro-inflammatory factors in brain tissue 12 h upon restoration of MCA perfusion was evaluated by quantitative real-time RT-PCR (mean ± SD; *n* = 3/3; Two-way ANOVA with Holm-Sidak’s multiple comparisons test). S, sham; M, MCAO. * *p* < 0.05

Gene set enrichment analyses on our whole genome expression data revealed a down-regulation of gene sets associated with inflammation and immune cell functions in the infarcted cerebral tissue of *Ephb2*^*−/−*^ mice (Table 1, Fig. 2). Given that brain tissue protection in *Ephb2*^*−/−*^ mice is already detectable during the first 6 h of a stroke, we reasoned that EphB2-dependent signaling might be involved in immediate early post-ischemic pro-inflammatory processes. Immunofluorescence techniques revealed an increasing number of neutrophils infiltrating the ipsilateral hemisphere from 6 to 24 h upon onset of reperfusion, whereas the frequency of neutrophils located at the non-affected brain hemisphere remained unchanged as compared to sham-operated animals (Fig. 4a; Additional file 1: Figure S4). The early post-ischemic recruitment of neutrophils into the brain of *Ephb2*^*−/−*^ mice was not significantly different from WT mice (Fig. 4a). In response to an ischemic insult, resident microglia and astrocytes localized in close vicinity to the infarct lesion site undergo cellular activation that causes morphological and immunophenotypic changes, enhanced proliferation, and increased production and release of cytokines, chemokines, and ROS [22]. Accordingly, as compared to sham-operated control animals, the number of both microglia/macrophages (Fig. 4b; Additional file 1: Figure S4) and astrocytes (Fig. 4c; Additional file 1: Figure S4) within the peri-infarct region gradually increased from 24 to 72 h post-reperfusion, but the quantities of microglia/macrophages and astrocytes across the peri-infarct zone of *Ephb2*^*−/−*^ mice were quite similar to those in WT littermates (Fig. 4b, c). Unfortunately, Iba1 did not allow distinguishing activated from resting microglia. At 12 h post-reperfusion, prior to any substantial accumulation of activated glial cells along the infarct border zone, we determined a markedly increased expression of key pro-inflammatory factors such as *Mcp-1, Il-1beta*, *Il-6*, and *Cox-2,* which was significantly less pronounced in the ipsilesional brain tissue of *Ephb2*^*−/−*^ mice in comparison to WT animals (Fig. 4d).

### EphB2/ephrin-B reverse signaling promotes pro-inflammatory activation of astrocytes through activation of NF-κB

**Fig. 5:**
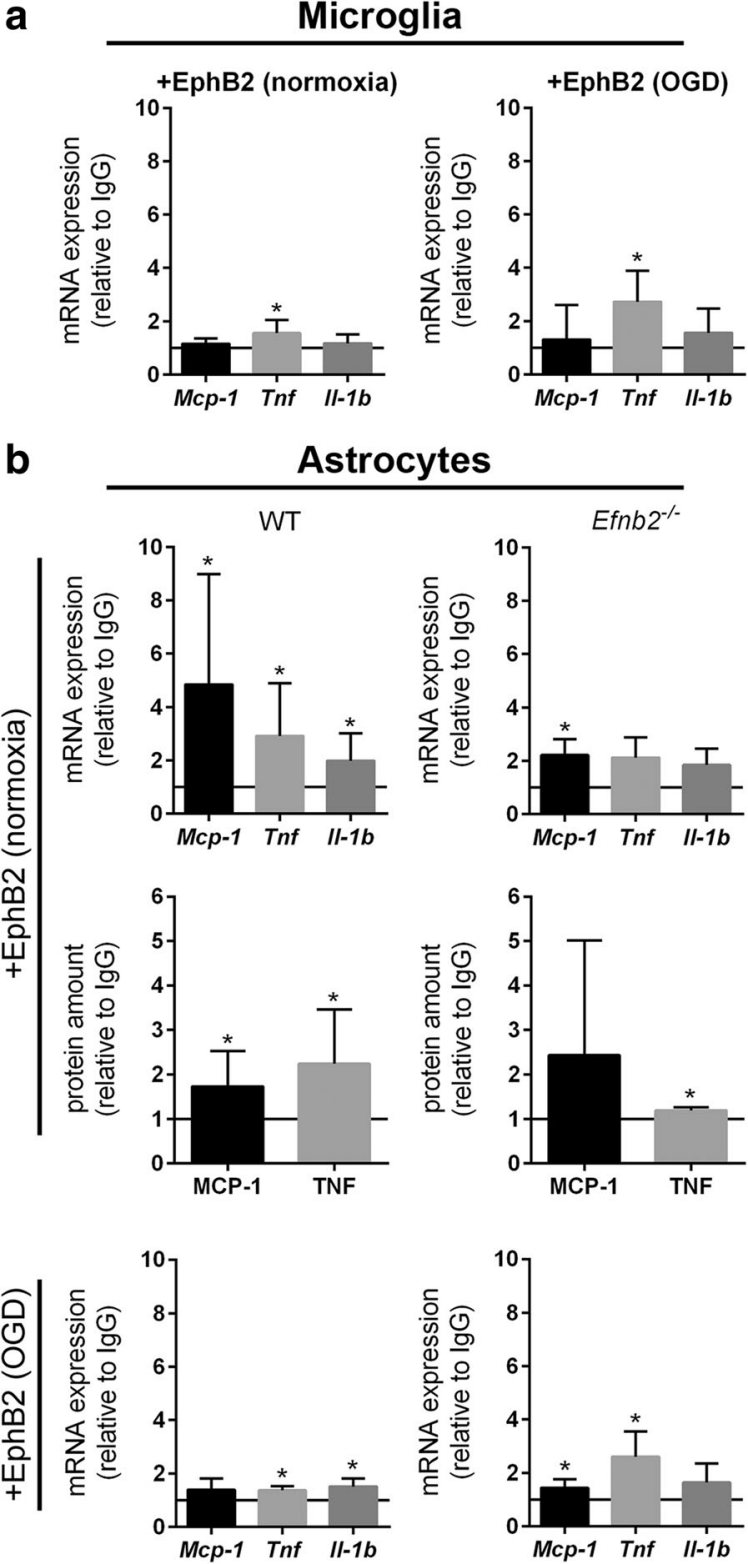
EphB2/ephrin-B reverse signaling promotes pro-inflammatory activation of astrocytes. **a** Primary microglia isolated from brains of neonatal WT mice were exposed to normoxic or OGD conditions for 6 h in the presence of either 10 nmol pre-clustered EphB2/Fc or anti-IgG Fc. Gene expression was determined by quantitative real-time RT-PCR (mean ± SD; *n* = 5 (N), *n* = 4 (OGD); Student’s *t*-test). **b** Primary astrocytes derived from brains of new born WT and *nEfnb2*^*Δ/Δ*^ mice were exposed to normoxic or OGD conditions for 6 h in the presence of either pre-clustered EphB2/Fc or anti-IgG Fc. Gene expression was determined by quantitative real-time RT-PCR (mean ± SD; *n* = 6/4 (N), *n* = 3/4 (OGD); Student’s *t*-test) and ELISA (mean ± SD; *n* = 12/9 (N); Student’s *t*-test). * *p* < 0.05

The above-mentioned results prompted us to study the role of EphB2/ephrin-B signaling in the inflammatory activation of microglia and astrocytes. Primary murine microglia, which were isolated from cortices of WT mice and exposed to either oxygen-glucose deprivation (OGD) or basal conditions in vitro, responded to stimulation with pre-clustered recombinant EphB2/Fc fusion proteins with a transcriptional up-regulation of *Tnf*, whereas the expression of *Mcp-1* and *Il-1beta* remained unchanged (Fig. 5a). On the contrary, the treatment with pre-clustered ephrin-B1 and ephrin-B2, two ligands of EphB2, did not affect inflammatory factor expression of non-stressed microglia (Additional file 1: Figure S5a). In microglia faced with OGD stress, ephrin-B2 increased the expression of *Il-1beta* (Additional file 1: Figure S5a). The phagocytic activity of both OGD- and non-stressed microglia was not altered by either EphB2, ephrin-B1 or ephrin-B2 (Additional file 1: Figure S5b). To further clarify the impact of EphB2/ephrin-B forward signaling on microglial activation, microglia isolated from the cortices of *Ephb2*^*−/−*^ mice were treated with ephrin-B1 or ephrin-B2. Neither the phagocytic activity nor the expression of pro-inflammatory factors of *Ephb2*^*−/−*^ microglial cells exposed to ischemic or control conditions were markedly affected upon treatment with ephrin-B1 or ephrin-B2 (Additional file 1: Figure S5a, b). Overall, our results do not point to an important role of EphB2/ephrin-B forward or reverse signaling for the activation of resident microglia during early acute ischemic stroke.

**Fig. 6:**
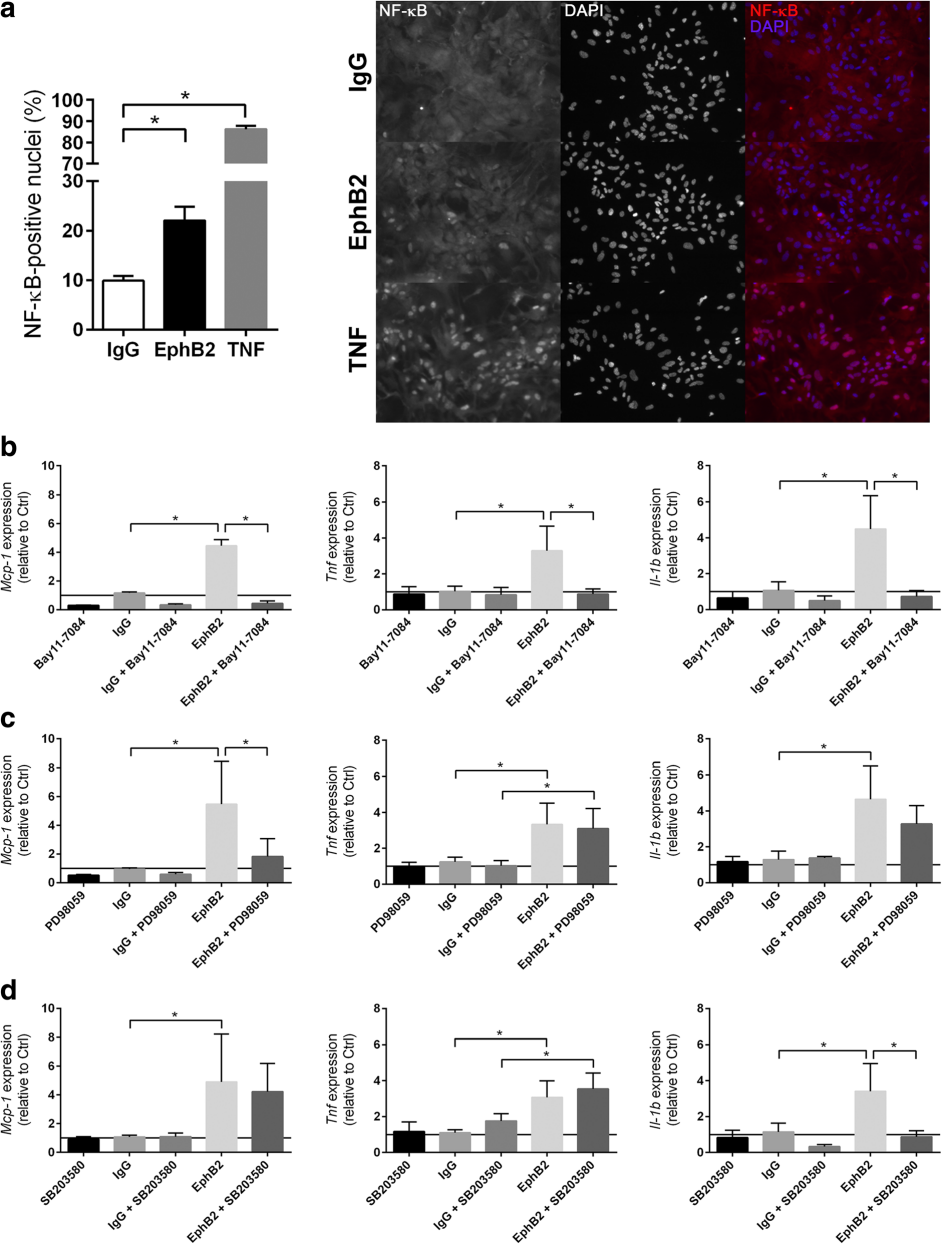
EphB2 promotes NF-κB-dependent pro-inflammatory activation of astrocytes through activation of Erk and p38-MAPK signaling cascade. **a** Astrocytes isolated from brains of neonatal WT mice were treated with 10 nmol pre-clustered EphB2/Fc, anti-IgG Fc or 30 nmol rmTNF for 6 h. Immunofluorescent staining was used to determine the nuclear accumulation of NF-κB (mean ± SD; *n* = 3; Student’s *t*-test). The top-right panel shows representative immunofluorescent staining images: NF-κB (red) and nuclei (blue). **b-d** Astrocytes were treated with either (**b**) 10 μM Bay 11–7082, (**c**) 20 μM PD98059 or (**d**) 10 μM SB203580 for 1 h prior to stimulation with pre-clustered EphB2/Fc or anti-IgG Fc for 6 h. Gene expression was analyzed by quantitative real-time RT-PCR (mean ± SD; *n* = 3 (Bay 11–7082), *n* = 3 (PD98059), *n* = 4 (SB203580); One-way ANOVA with Holm-Sidak’s multiple comparisons test). * *p* < 0.05

Primary murine astrocytes isolated from brain tissue of WT mice showed significantly increased mRNA levels of *Mcp-1*, *Tnf* and *Il-1beta* upon treatment with EphB2 (reverse signaling, Fig. 5b). A pro-inflammatory response to EphB2, albeit to a lesser extent, was also observed in astrocytes subjected to OGD (Fig. 5b). Astrocytic up-regulation of MCP-1 and TNF upon EphB2 treatment was further confirmed at the protein level (Fig. 5b). By contrast, ephrin-B1- or ephrin-B2-induced forward signaling did not elevate inflammatory factor expression of either OGD- or non-stressed astrocytes (Additional file 1: Figure S5c). In *Ephb2*^*−/−*^ astrocytes (Additional file 1: Figure S6a) cultured under basal conditions, ephrin-B2, but not ephrin-B1, reduced the expression of *Mcp-1*, *Tnf*, and *Il-1beta*. However, the anti-inflammatory transcriptional response towards ephrin-B2 was not detectable in *Ephb2*^*−/−*^ astrocytes subjected to OGD (Additional file 1: Figure S5c). Next, we investigated whether EphB2-induced reverse signaling is mediated via the activation of astrocytic ephrin-B1, ephrin-B2 or both. EphB2 treatment of non-stressed cortical astrocytes isolated from transgenic mice having a deficiency for the *Efnb2* gene in cells of the neural lineage (Additional file 1: Figure S6b) failed to up-regulate *Tnf* and *Il-1beta*, while transcription of *Mcp-1* was still significantly increased, albeit to a lesser extent as compared to WT astrocytes (Fig. 5b). Exposure to OGD stress enhanced the responsiveness of *Efnb2*^*−/−*^ astrocytes for EphB2 and resulted in significant up-regulation of *Mcp-1* and *Tnf* (Fig. 5b). Thus, our findings suggest that EphB2-induced reverse signaling through both ephrin-B1 and ephrin-B2 drives the pro-inflammatory activation of astrocytes. In an attempt to unravel the molecular basis underlying the EphB2-mediated inflammatory activation of astrocytes, we demonstrated that EphB2 favors the nuclear translocation of NF-κB, well known to promote the expression of pro-inflammatory factors at the transcriptional level (Fig. 6a). Accordingly, pre-treatment with BAY 11–7082, a potent inhibitor of the IκB kinase (key upstream regulator of NF-κB), completely prevented the EphB2-induced up-regulation of *Mcp-1*, *Tnf*, and *Il-1beta* (Fig. 6b). Moreover, pharmacological inhibition of mitogen-activated protein kinase kinase 1 and 2 (MAP2K1/2), which phosphorylates extracellular-signal-regulated kinase 1 and 2 (ERK1/2) and p38 MAPK, prior to EphB2 stimulation prevented transcriptional up-regulation of *Mcp-1* (Fig. 6c) and *Il-1beta* (Fig. 6d), respectively. In contrast, inhibition of Src-family kinases (Additional file 1: Figure S7a), c-Jun N-terminal kinase (JNK; Additional file 1: Figure S7b) or phosphoinositide 3-kinase (PI3K; Additional file 1: Figure S7c), which have also been described as downstream targets of phosphorylated ephrin-B1 or -B2 and activators of NF-κB, did not impede EphB2-induced up-regulation of either *Mcp-1*, *Tnf*, or *Il-1beta* (Additional file 1: Figure S7a-c). We further analyzed whether ischemic stress conditions affect the expression of ephrin-B1 and ephrin-B2 in glial cells, and thus might alter their responsiveness toward EphB2. Indeed, exposure of microglia and astrocytes to ischemic or pure hypoxic conditions significantly increased the mRNA expression of *Efnb2*, while the transcript levels of *Efnb1* and *Ephb2* remained unchanged as compared to control conditions (Additional file 1: Figure S8a). Given that reduction of the cellular oxygen level was sufficient to up-regulate glial ephrin-B2, we investigated the possibility that *Efnb2* is transcriptionally regulated through hypoxia-inducible factor (HIF). Cells were treated with dimethyloxallyl glycine (DMOG), an inhibitor of the prolyl 4-hydroxylase domain proteins (PHD), which act as key suppressors of the HIF pathway. While astrocytes responded to DMOG with a strong up-regulation of the classical HIF target gene *Vegfa*, the mRNA levels of *Efnb2* remained stable over time (Additional file 1: Figure S8b). The latter suggests that the PHD-HIF axis is not responsible for the oxygen-dependent *Efnb2* gene expression. However, in ipsilesional brain tissue of mice subjected to I/R injury, neither *Efnb2* nor *Efnb1* or *Ephb2* were found to be differently expressed compared to levels measured in cerebral tissue from sham-treated animals (Additional file 1: Figure S8c).

Collectively, our results indicate that EphB2-mediated activation of both ephrin-B1 and ephrin-B2 promotes the NF-κB-dependent pro-inflammatory activation of astrocytes, but not microglia, through activation of MAPK signaling.

### EphB2 deficiency prevents NMDAR-evoked excitotoxic mitochondrial depolarization in neurons

Excessive release of glutamate into the extracellular space and subsequent overactivation of NMDARs is a crucial step in the ischemic cascade [10]. Given the reduced neuronal cell death and extent of acute cytotoxic edema in *Ephb2*-deficient mice (Figs. 1c, 3c, d) upon ischemic stroke, we hypothesized that in this context EphB2 activation (Fig. 1b) contributes to neuronal excitotoxicity. In light of the importance of NMDAR-mediated excitotoxicity during cerebral ischemia [28], we consequently aimed at analyzing the function of NMDARs in *EphB2-*deficient neurons in the context of excitotoxicity.

**Fig. 7:**
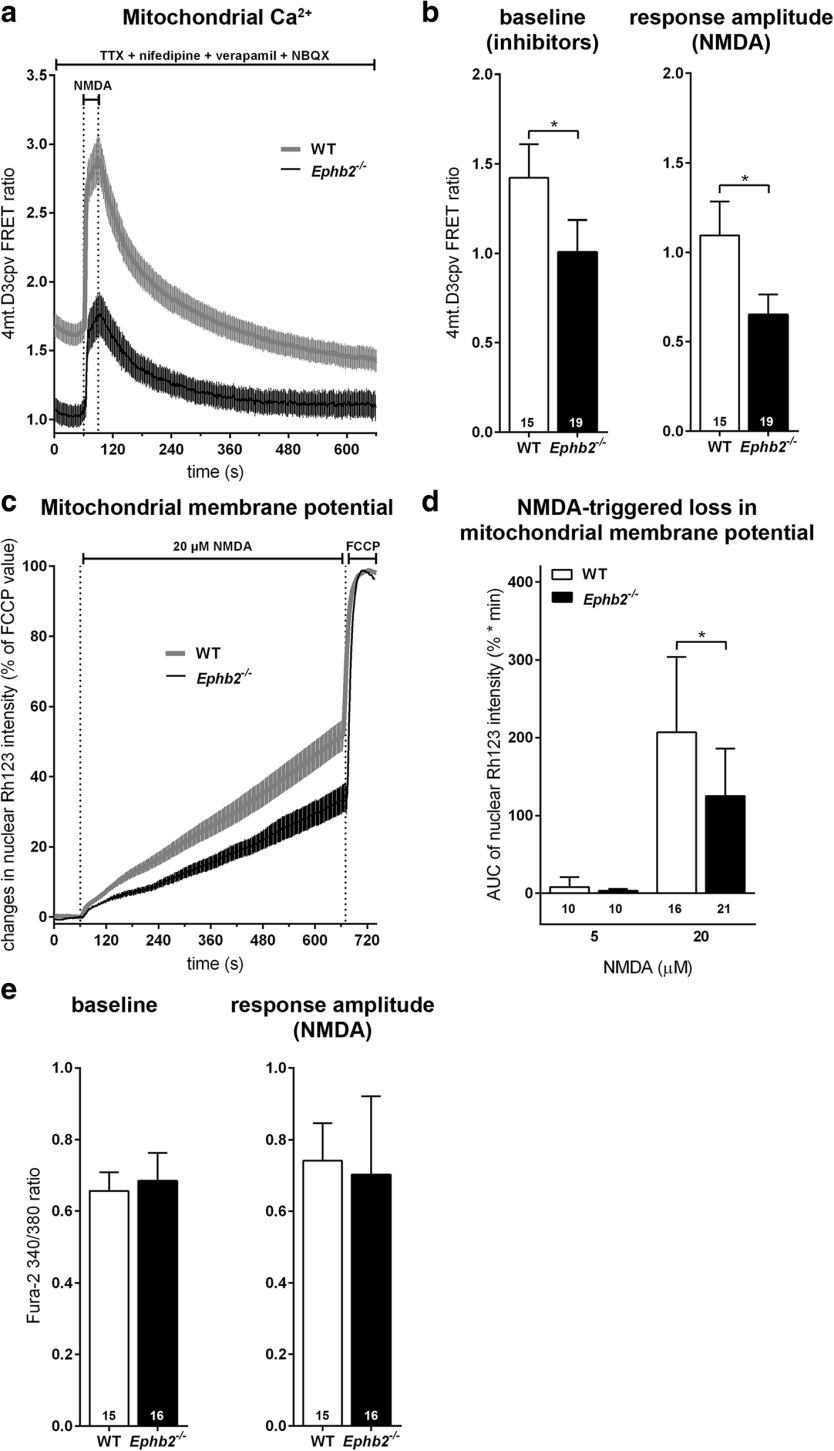
EphB2 deficiency inhibits NMDAR-dependent mitochondrial Ca^2+^ responses and mitochondrial membrane depolarization in neurons. WT and *Ephb2*^*−/−*^ forebrain neurons were obtained from P0 mice. **a**, **b** Mitochondrial calcium imaging using the FRET-based indicator 4mt.D3cpv was performed with drugs in the bath to inhibit voltage-dependent calcium channels, AMPA receptors, and voltage-dependent sodium channels. This should prevent APs and associated voltage-dependent calcium signals, leaving the “pure” NMDA signal, which was evoked by a brief (30 s) application of 20 μM NMDA. **a** Representative data from one coverslip each of WT and *Ephb2*^*−/−*^ cells showing the baseline 4mt.D3cpv FRET ratio in the presence of inhibitors and the response to NMDA (mean ± SEM). **b** Quantification of the baseline 4mt.D3cpv FRET ratio and peak amplitude of the response to NMDA (mean ± SD; *n* = 15/19 coverslips from 4 independent preparations; Student’s *t*-test). **c**, **d** Mitochondrial membrane potential imaging using the fluorescent dye Rh123. Under basal conditions, Rh123 accumulates within the mitochondrial matrix, where its high concentration leads to quenching. Mitochondrial membrane depolarization induces leakage of Rh123 from the mitochondria into the cytoplasm, where its fluorescence is dequenched resulting in an increase in fluorescence intensity. Rh123 fluorescence levels were measured in the nucleus to avoid possible contamination by fluorescence signals emerging from mitochondria. **c** Representative data from one coverslip each of WT and *Ephb2*^*−/−*^ cells during stimulation with 20 μM NMDA over ~ 10 min (mean ± SEM). **d** Quantification of the area under the curve during the first 9 min of NMDA treatment for 5 and 20 μM NMDA (mean ± SD; *n* = 10 coverslips from 4 independent preparations (5 μM), *n* = 16/21 coverslips from 4 independent preparations (20 μM); Student’s *t*-test). **e** Quantification of cytoplasmic Ca^2+^ levels under baseline conditions and the peak amplitude of the cytoplasmic Ca^2+^ response as measured using the ratiometric small molecule indicator fura-2 triggered by brief (30 s) stimulation with 20 μM NMDA (mean ± SD; *n* = 15/16 coverslips from 4 independent preparations; Student’s *t*-test). * *p* < 0.05

To this end, primary cortical cultures, consisting of > 80% neurons, were isolated from brains of newborn (P0) WT and *Ephb2*^*−/−*^ mice, and cultured for ten days in vitro (DIV10) to achieve the development of an extensive network of synaptic contacts [53], and proper susceptibility to NMDA-induced excitotoxicity [12]. Along with this, we confirmed that DIV10 neurons express EphB2 protein on the cell surface (Additional file 1: Figure S9). Cytoplasmic and mitochondrial Ca^2+^ levels, as well as changes in mitochondrial membrane potential, were evaluated before and during NMDAR stimulation. In order to prevent action potentials (APs) and associated Ca^2+^ signals not mediated through NMDAR activation, neurons were treated with drugs, which inhibit voltage-dependent Ca^2+^ channels, AMPA receptors (AMPARs), and voltage-dependent sodium channels. Those treatments already caused a decrease of baseline mitochondrial Ca^2+^ levels, assessed as a decrease in the baseline 4mt.D3cpv FRET ratio, in *Ephb2*^*−/−*^ neurons. Further, the NMDA-triggered increase in mitochondrial Ca^2+^ levels was significantly reduced in *Ephb2*^*−/−*^ neurons when compared to WT neurons (Fig. 7a, b). This suggests that mitochondrial Ca^2+^ homeostasis regulated by NMDARs is impaired already under baseline conditions when EphB2 is absent and that neurons are protected from excitotoxic mitochondrial Ca^2+^ overload by the lack of EphB2.

As extrasynaptic NMDAR stimulation is known to promote cell death via breakdown of the mitochondrial membrane potential, the next experiments aimed at identifying whether and how the absence of EphB2 may influence mitochondrial membrane potential responses to NMDAR stimulation. Cells were loaded with the fluorescent dye Rhodamine 123 (Rh123). Exposing neurons to the mitochondrial uncoupler FCCP results in maximum fluorescence intensity of Rh123. Neurons were stimulated with NMDA and changes in Rh123 fluorescence intensity, expressed as % of the FCCP-evoked maximum, were quantified. Stimulation with low-dose NMDA did not lead to changes in Rh123 fluorescence and did not reveal any differences between the two genotypes. However, when cells were treated with high-dose NMDA, *Ephb2*^*−/−*^ neurons showed a significantly smaller increase in Rh123 intensity when compared to WT neurons indicating that *Ephb2*^*−/−*^ neurons are less susceptible to the NMDA-induced mitochondrial membrane depolarization that is associated with mitochondrial Ca^2+^ overload during an excitotoxic stimulus (Fig. 7c, d).

Ca^2+^ imaging using the ratiometric dye fura-2 was performed to examine global cytoplasmic Ca^2+^ levels at baseline and during selective stimulation of NMDARs as above. Neither baseline nor NMDA-stimulated cytoplasmic Ca^2+^ rises were different between the two genotypes. These results indicate that NMDAR-mediated cytoplasmic Ca^2+^ signaling is not affected upon loss of EphB2 (Fig. 7e).

Synaptic activity can trigger a nuclear Ca^2+^-driven neuroprotective gene program leading to a reduction in excitotoxicity-associated mitochondrial Ca^2+^ load [9, 59]. Consequently, neurons are less sensitive to excitotoxic cell death and ischemia [9, 51, 58]. AP bursting was triggered by treatment with the type A gamma-aminobutyric acid receptor (GABAaR) antagonist gabazine, and cytoplasmic and mitochondrial Ca^2+^ responses were measured with Fura-2 and 4mt.D3cpv, respectively. The amplitudes of cytoplasmic Ca^2+^ responses to a single burst of APs were attenuated in *Ephb2*^*−/−*^ neurons (Additional file 1: Figure S10a). However, mitochondrial Ca^2+^ rises in response to an AP burst were not different between genotypes (Additional file 1: Figure S10b, c).

Taken together, our imaging data show both attenuated NMDAR-dependent mitochondrial Ca^2+^ signaling and diminished sensitivity of the mitochondrial membrane potential to NMDAR stimulation in *Ephb2*^*−/−*^ neurons. These findings support the hypothesis that EphB2 deficiency protects neurons from NMDAR-induced excitotoxicity.

### Ephrin-B2 deficiency reduces brain tissue injury in mice suffering from acute ischemic stroke

**Fig. 8:**
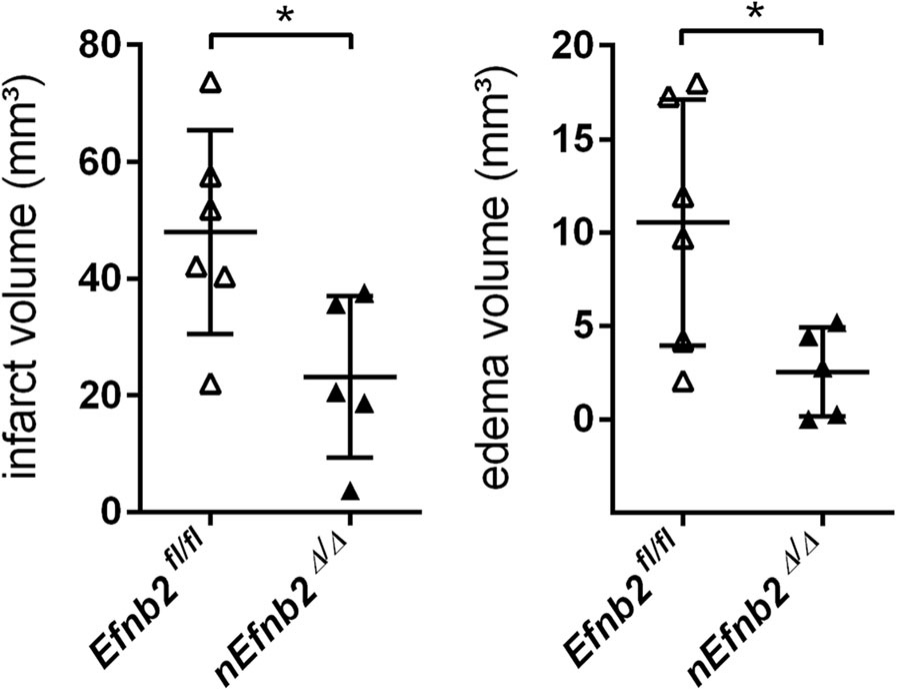
Ephrin-B2 deficiency reduces brain tissue injury in mice suffering from acute ischemic stroke. *Efnb2*^*fl/fl*^ and *nEfnb2*^*Δ/Δ*^ mice were subjected to 60 min MCAO followed by 24 h of reperfusion. Brain sections were stained with cresyl violet, and infarct and edema sizes were analyzed using ImageJ. Infarct volume is edema-corrected (single values and mean ± SD; *n* = 6/5; Student’s *t*-test). * *p* < 0.05

The early phosphorylation of EphB2 (Fig. 1b) after the onset of ischemia, prompted us finally to have a closer look at its ligand ephrin-B2. Thus, we analyzed whether conditional ablation of the *Efnb2* gene in cells of the neural lineage may result in a similar protection of the murine CNS from acute ischemic stroke as indicated for *Ephb2*^*−/−*^ mice (Fig. 1c-f). In fact, *nEfnb2*^*Δ/Δ*^ mice showed a significantly decreased infarct lesion size and brain swelling in comparison to *Efnb2*^*fl/fl*^ littermates (Fig. 8).

Overall, our experimental data strongly suggest that glutamate-induced excitotoxic neuronal damage and inflammation during early acute ischemic stroke is substantially enhanced by EphB2/ephrin-B2 forward and reverse signaling in neurons and astrocytes, respectively.

## Discussion

Several previous studies in rodent models of cerebral ischemia have demonstrated that EphB/ephrin-B signaling is important for the regulation of delayed endogenous adaptive processes such as neurogenesis [11, 55] and angiogenesis [14, 56], and as such promotes long-term recovery from ischemic stroke. Whether or not EphB/ephrin-B family members are also involved in acute events during the pathogenesis of stroke is, however, entirely unknown. To the best of our knowledge, we here provide the first experimental evidence that EphB2-dependent signaling is crucial for certain pathophysiological processes that are initiated in the early acute phase after ischemic stroke. The major novel findings of the present study include the following: (1) loss of EphB2 function diminishes infarct size, brain swelling and functional impairments in the early acute stage of ischemic stroke. (2) Acute inflammatory gene expression in response to cerebral ischemia is reduced in *Ephb2*-deficient mice. On the cellular level, EphB2/ephrin-B reverse signaling drives NF-κB- and MAPK-dependent pro-inflammatory activation of resident astrocytes. (3) The extent of cytotoxic edema developing in the infarct core within the first few hours after stroke onset was strongly decreased in *Ephb2*-deficient brains. Accordingly, neurons lacking EphB2 show a reduced vulnerability to mitochondrial dysfunction in the course of NMDA-induced excitotoxicity.

On the mechanistic level, the observed decrease in infarct size in *Ephb2*-deficient mice may originate from an alteration of multiple cellular and molecular interactions as EphB/ephrin signaling may influence the function of different cell types belonging to the vascular, immune or neuronal system. In fact, with respect to the vascular unit, a putative impact of EphB2 on the cerebrovascular anatomy and functionality is deduced from studies suggesting that mesenchymal EphB2 is required to communicate with vascular endothelial ephrin-B-ligands for adequate blood vessel development [1]. Moreover, ephrin-B2/EphB4 signaling has been shown to be involved in the restoration of the BBB and neurovascular repair mechanisms upon cerebral ischemia [14], underlining the importance of ephrin/Eph communication at the neurovascular interface for cellular responses to stroke. However, our study revealed that neither cerebrovascular anatomy, capillary density, and pericyte coverage, nor the reduction in rCBF upon MCAO was altered in *Ephb2*-deficient as compared to WT mice suggesting that loss of EphB2 does not directly affect the phenotype of vascular cells in response to cerebral ischemia. Additionally, we investigated the functional responses of the vascular unit and analyzed the development of vasogenic edema that evolves from an increase in permeability of the BBB thereby allowing extravasation of fluid into the brain parenchyma [13]. However, as assessed by immunofluorescence-, dye extravasation-, and MRI-based analyses, vascular gap formation, BBB hyperpermeability, and vasogenic edema formation were comparable in mutant and WT mice within the first 6 h upon cerebral ischemia. Collectively, none of our findings supports a primary role of vascular EphB2 on infarct and edema formation.

Another aspect of the EphB/ephrin-B system is its capacity to modulate immune responses, a feature which may provide an alternative explanation for the observed less severe stroke response of *Ephb2*-deficient mice. In fact, EphB6 deficient mice show an impaired T-cell function [35, 36], and ephrin-B1 and ephrin-B2 are required to retard the internalization of IL-7Rα from the T-cell surface [34]. Furthermore, monocytes express several types of EphB receptors [5, 26, 41] and upregulate EphB2 expression upon activation [5, 45] to allow for binding of compatible ephrin-B ligands promoting the release of MCP-1 and IL-8 [5]. Here, we show that the early (12 h) expression of prototypic mediators of I/R-associated pro-inflammatory responses such as MCP-1 or IL-6 was decreased in *Ephb2*-deficient mice while the infiltration of microglia/macrophages and astrocytes into the peri-infarct region was not altered. Mechanistically, especially the pro-inflammatory activation of astrocytes may be supported under these conditions by triggering an EphB2/ephrin-B reverse signaling to stimulate NF-κB-mediated cytokine expression via the MAPK pathway as evidenced by our in vitro findings. Interestingly, a recent report indicates that astrocytic ephrin-B1 reverse signaling induced by its interaction with the neuronal EphB receptor is responsible for synapse engulfment [25]. Along these lines, it is tempting to speculate that loss of synaptic EphB2 may also affect ephrin-B1-dependent astrocyte activation after cerebral injury. However, whether the attenuated inflammatory response we observed is a direct consequence of an environment devoid of EphB2 receptors or is indirectly caused by the reduced number of neurons dying due to excitotoxicity cannot be fully elucidated based on the chosen experimental setup.

Cytotoxic brain edema triggered by neuronal swelling is the main cause of mortality following cerebral ischemia [44]. Neuronal swelling owing to ischemic stress is rapidly initiated by aberrant depolarization and entry of sodium and chloride ions, primarily caused by reduced activity of the Na^+^/K^+^ ATPase and accumulation of extracellular glutamate due to synaptic release/spillover, impairment or reversal of uptake mechanisms, and activation of voltage-dependent channels. The resultant increase of cytoplasmic sodium and chloride causes an osmotic imbalance that leads to water entry and cytotoxic edema contributing to neuronal cell death within minutes to hours upon cerebral ischemia [44]. Our MRI studies revealed a reduced cytotoxic edema in the infarct core of *Ephb2*-deficient mice already 6 h after cerebral ischemia, and thus point to a potential relationship between neuronal EphB2 function and the extent of glutamate excitotoxicity during ischemic stroke. NMDARs, glutamate and voltage-gated ion channels permeable for calcium, are central for the pathological processes that underlie glutamate excitotoxicity [28]. In addition to their synaptic localization, NMDARs are found at extrasynaptic sites. The subunit composition of the receptors within and outside synaptic contacts is similar, although, in addition to carrying the common GluN1 subunit, extrasynaptic NMDARs contain preferentially the GluN2B subunit, whereas GluN2A is the predominant subunit in synaptic NMDARs [2]. Paradoxically, activation of synaptic NMDARs induces neuroprotective mechanisms, whereas stimulation of extrasynaptic NMDARs promotes neuronal cell death. This difference results from the activation of distinct genomic programs and from opposing actions on intracellular signaling pathways [2, 19, 20]. Thus, any shift in balance to reduce synaptic or enhance extrasynaptic NMDAR signaling may be detrimental to neuronal viability [39]. Calcium overload-induced mitochondrial dysfunction is a central component in the glutamate-evoked excitotoxic injury of CNS neurons [47]. It is noteworthy to mention that extrasynaptic NMDARs may be in close contact with mitochondria, whereas the postsynaptic scaffold, and also the spine structure as such, keeps mitochondria at a distance to synaptic NMDARs [2]. Thus, upon stimulation of extrasynaptic, but not synaptic NMDA receptors, mitochondria are exposed to high and possibly damaging calcium rises [2]. Interestingly, several previous studies clearly demonstrated that EphB2 controls the assembly, localization and activity of NMDARs in neurons. On a mechanistic level, the ephrin-B-induced phosphorylation of a single tyrosine (Y504) in the extracellular domain of EphB2 leads to direct recruitment of NR1 and its associated subunits NR2A and NR2B to EphB2 [8, 16, 17]. Along with binding and clustering of NMDARs, ephrin-B2-mediated activation of EphB2 also enhances glutamate-stimulated Ca^2+^ influx through the NMDAR [50]. The latter requires the cytoplasmic tyrosine kinase domain of EphB2 recruiting and activating Src family members, which in turn phosphorylate NR2B at Y1472, which itself is of crucial importance for the synaptic localization and retention of NMDARs [38, 50]. Thus, it is tempting to speculate that neuronal EphB2 may be a positive regulator of extrasynaptic NMDARs. In fact, our own work revealed that loss of EphB2 in post-natal neurons selectively diminished mitochondrial Ca^2+^ load upon activation of NMDAR but not in response to AP bursting. On the molecular level, excessive Ca^2+^ influx into mitochondria decreases the electrochemical gradient across the mitochondrial membrane leading to reduced ATP synthesis, release of pro-apoptotic proteins, activation of calcium-dependent proteases and elevated ROS production [42]. Consistent with the inhibition of mitochondrial Ca^2+^ overload, which triggers mitochondrial dysfunction during glutamate excitotoxicity, mitochondrial membrane depolarization was less distinct in *Ephb2*-deficient neurons in comparison to WT neurons stimulated with NMDA. Along this line, we showed for the first time that EphB2 is rapidly activated in the CNS of mice suffering from cerebral ischemia. Moreover, brain-specific loss of ephrin-B2 in mice reduced the extent of cerebral tissue damage in the acute phase of ischemic stroke.

## Conclusion

Taken together, our experimental data indicate that EphB2 signaling (i) contributes to the excitotoxic NMDAR-dependent Ca^2+^ overload of mitochondria and subsequent mitochondrial membrane depolarization and cell death in neurons, and (ii) promotes the pro-inflammatory activation of astrocytes. These findings indicate an important role of the EphB/ephrin-B system during acute ischemic stroke. Further, our results suggest that inhibition of EphB2 may specifically attenuate extrasynaptic NMDAR function, and thereby improve stroke outcome.

Although NMDAR-mediated excitotoxicity is a central feature of neuronal cell death in stroke as well as in other neurodegenerative diseases, NMDARs are also important for cell survival and plasticity [39]. Recent data suggest that this dual role for neuronal survival and death depends on the subcellular localization and subunit composition of NMDARs [2, 54]. Although all clinical trials aimed at blockade of NMDAR function to treat stroke patients failed, new hope comes from studies focusing on death-signaling pathways downstream from extrasynaptic NMDARs [54]. As a new therapeutic approach, we here suggest to specifically attenuate detrimental extrasynaptic NMDAR activity by inhibition of the EphB/ephrin-B system. This approach might shift the balance towards pro-survival signals mediated through synaptic NMDAR-induced processes [2].

## Additional files

Additional file 1: **Figure S1.** Characterization of *Ephb2* and *Efnb2* gene ablation in brain tissue and purity of primary cell cultures. **Figure S2.** EphB2 promotes brain tissue damage during early acute ischemic stroke in a gene dosage-dependent manner. **Figure S3.** EphB2 deficiency does not affect anatomy of the cerebral vascular system. **Figure S4.** Immunofluorescent detection of peripheral and resident immunocompetent cells in the CNS after acute stroke. **Figure S5.** EphB2/ephrin-B forward signaling does not induce pro-inflammatory activation of microglia and astrocytes. **Figure S6.** Characterization of *Ephb2* and *Efnb2* gene ablation in primary astroglial cultures. **Figure S7.** Pro-inflammatory activation of astrocytes by EphB2 does not require Src-, JNK- or PI3K-dependent signaling pathways. **Figure S8.** Hypoxic or ischemic stress leads to HIF-independent up-regulation of ephrin-B2 in glial cells. **Figure S9.** Primary murine neurons are a source of EphB2 surface proteins. **Figure S10.** Cytoplasmic and mitochondrial Ca^2+^ levels during AP bursting. WT and *Ephb2*^*−/−*^ forebrain neurons were obtained from P0 mice. (PDF 1786 kb)

Additional file 2: **Table S1.** List of primers used to genotype mice. **Table S2.** Overview of animals that met defined exclusion criteria. **Table S3.** Primary antibodies used for immunofluorescent staining. **Table S4.** List of primers used for quantitative real-time RT-PCR. **Table S5.** KEGG pathway-Based gene set enrichment analyses (GSEA). (PDF 271 kb)

Additional file 3 Supplementary Methods. (PDF 204 kb)
