# Supplementary material for: EphB2-dependent signaling promotes neuronal excitotoxicity and inflammation in the acute phase of ischemic stroke
Source: Acta Neuropathol Commun. 2019 Feb 5;7:15. doi: 10.1186/s40478-019-0669-7 (PMC6362601; doi:10.1186/s40478-019-0669-7)
Supplement: Supplementary file 1 — Figure S1. Characterization of Ephb2 and Efnb2 gene ablation in brain tissue and purity of primary cell cultures. Figure S2. EphB2 promotes brain tissue damage during early acute ischemic stroke in a gene dosage-dependent manner. Figure S3. EphB2 deficiency does not affect anatomy of the cerebral vascular system. Figure S4. Immunofluorescent detection of peripheral and resident immunocompetent cells in the CNS after acute stroke. Figure S5. EphB2/ephrin-B forward signaling does not induce pro-inflammatory activation of microglia and astrocytes. Figure S6. Characterization of Ephb2 and Efnb2 gene ablation in primary astroglial cultures. Figure S7. Pro-inflammatory activation of astrocytes by EphB2 does not require Src-, JNK- or PI3K-dependent signaling pathways. Figure S8. Hypoxic or ischemic stress leads to HIF-independent up-regulation of ephrin-B2 in glial cells. Figure S9. Primary murine neurons are a source of EphB2 surface proteins. Figure S10. Cytoplasmic and mitochondrial Ca2+ levels during AP bursting. WT and Ephb2−/− forebrain neurons were obtained from P0 mice. (PDF 1786 kb) [file 40478_2019_669_MOESM1_ESM.pdf]

ADDITIONAL FILE 1

EphB2-dependent signaling promotes neuronal excitotoxicity and inflammation in the acute phase of ischemic stroke

Anne-Sophie Ernst [1,*], Laura-Inès Böhler [1,*], Anna M. Hagenston [2], Angelika Hoffmann [3], Sabine Heiland [4], Carsten Sticht [5], Martin Bendszus [3], Markus Hecker [1], Hilmar Bading [2], Hugo H. Marti [1], Thomas Korff [1,*], Reiner Kunze [1,*]

[1] Institute of Physiology and Pathophysiology, Department of Cardiovascular Physiology, Heidelberg University, Heidelberg, Germany

[2] Department of Neurobiology, Interdisciplinary Center for Neurosciences, Heidelberg University, Heidelberg, Germany

[3] Department of Neuroradiology, Heidelberg University Hospital, Heidelberg, Germany

[4] Division of Experimental Radiology, Department of Neuroradiology, Heidelberg University Hospital, Heidelberg, Germany

[5] Center of Medical Research, Medical Faculty Mannheim, Heidelberg University, Heidelberg, Germany

[*] equal contribution

Address for correspondence:
Hugo H. Marti
Heidelberg University
Institute of Physiology and Pathophysiology
Im Neuenheimer Feld 326
69120 Heidelberg, Germany
Phone 49-6221-544138
FAX 49-6221-548224
E-mail hugo.marti@physiologie.uni-heidelberg.de

Supplementary Figures and Figure Legends

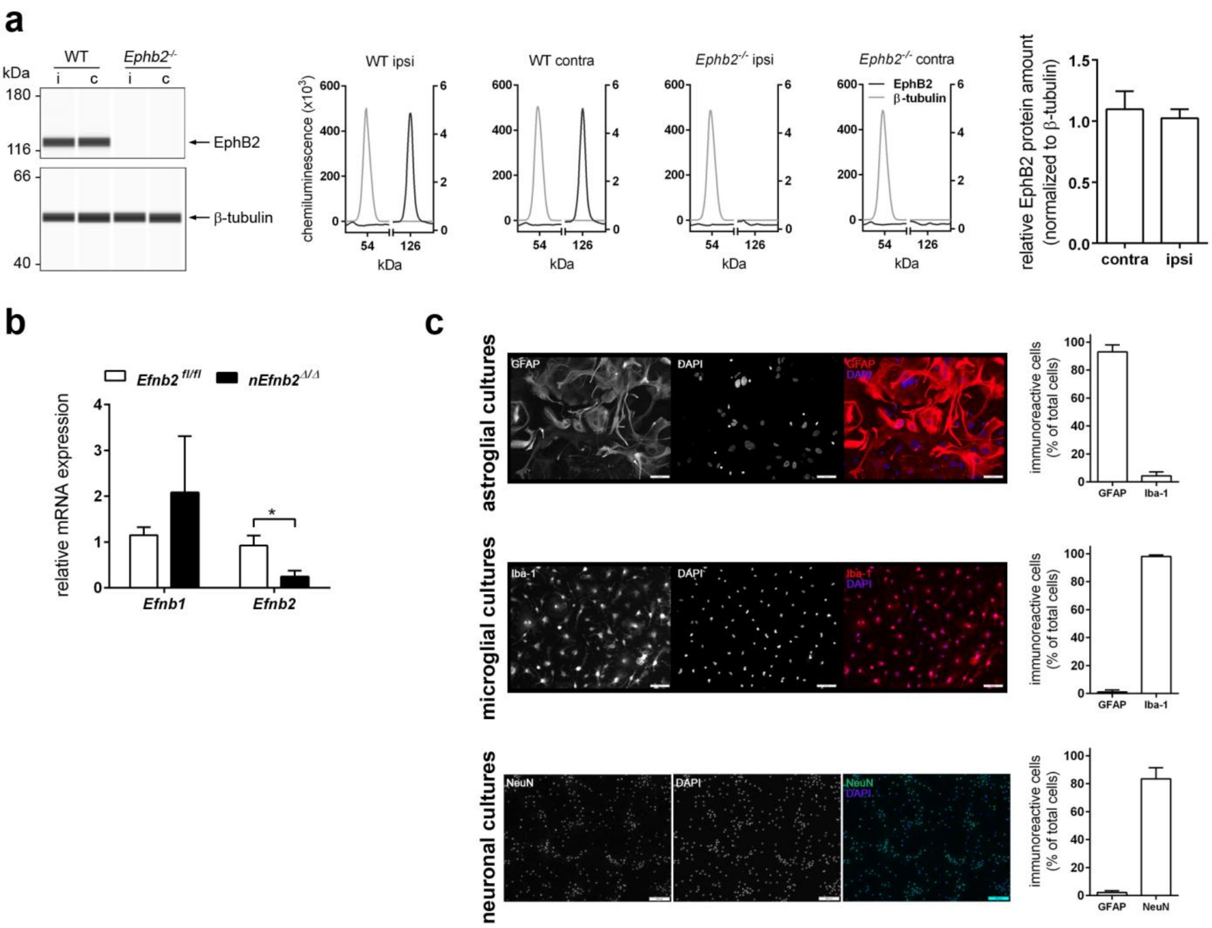

Fig. S1 Characterization of *Ephb2* and *Efnb2* gene ablation in brain tissue and purity of primary cell cultures. **(a)** WT and *Ephb2*$^{-/-}$ mice (*n*=3/group) were subjected to 60 min MCAO followed by 6 h of reperfusion. Protein lysates isolated from the contra- and ipsilateral brain hemisphere were analyzed for EphB2 abundance by capillary electrophoresis (Simple Western Wes-based technique). EphB2 protein amount in the contra- and ipsilateral brain tissue of WT mice normalized to beta-tubulin levels as loading control is plotted in the right bar graph (mean±SD; *n*=3; Student's *t*-test). **(b)** Expression of *Efnb1* and *Efnb2* in the forebrain of naïve *Efnb2*$^{fl/fl}$ and *nEfnb2*$^{\Delta/\Delta}$ mice was evaluated by quantitative real-time RT-PCR (mean±SD; *n*=4/6; Student's *t*-test). **(c)** Immunofluorescent staining of GFAP, Iba-1 and NeuN was applied to evaluate the purity of astroglial, microglial and neuronal cell cultures (*n*=3-5/group) originating from brains of neonatal mice. Representative immunofluorescent staining images: GFAP/Iba-1 (red), NeuN (green) and nuclei (blue). Scale bar = 50 or 100 µm.

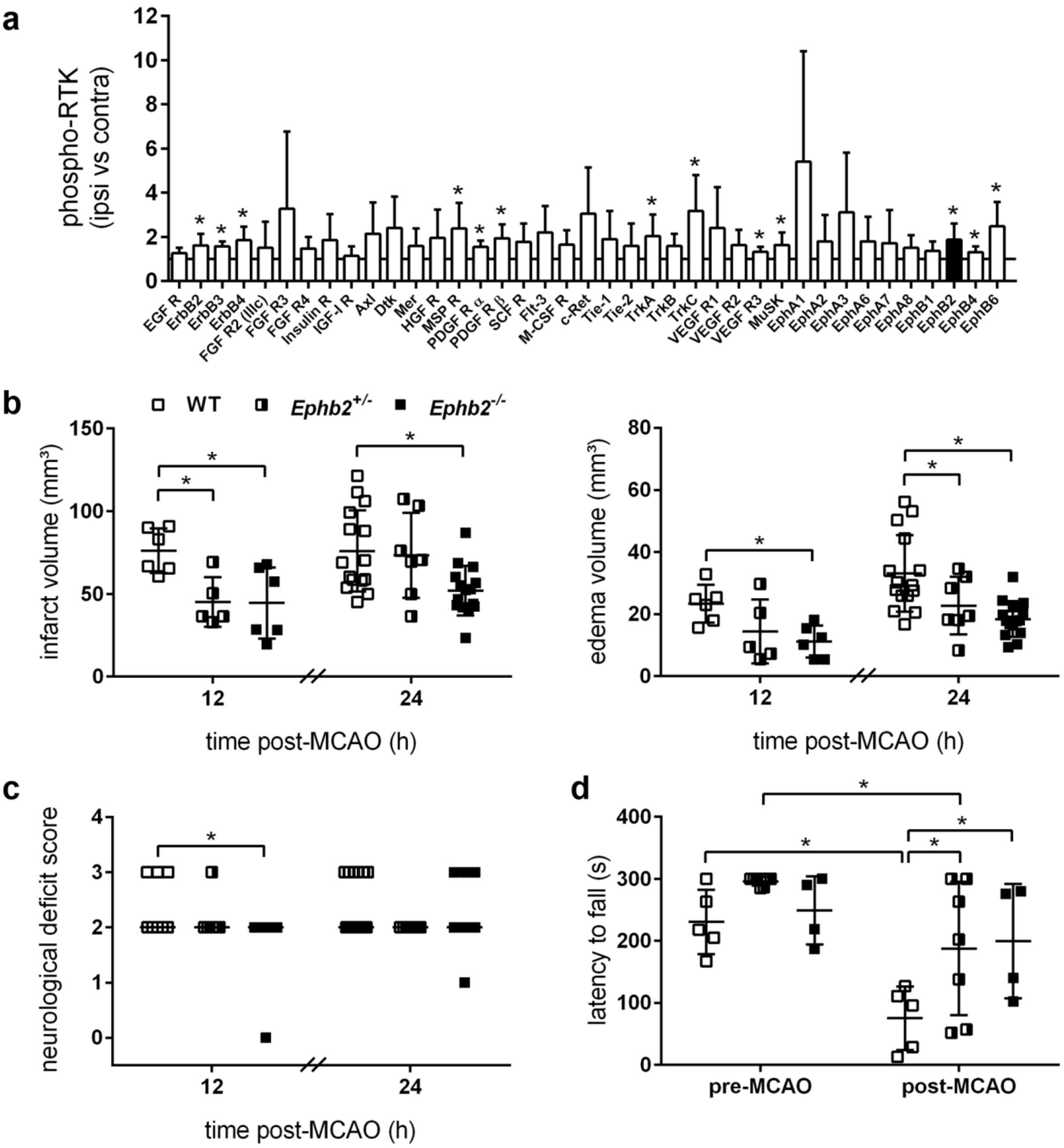

Fig. S2 EphB2 promotes brain tissue damage during early acute ischemic stroke in a gene dosage-dependent manner. **(a)** WT mice were subjected to 60 min MCAO followed by 6 h of reperfusion. Proteins were isolated from contra- and ipsilateral brain hemispheres and applied to proteome profiler membranes for detection of phosphorylation levels of receptor tyrosine kinases (mean±SD; *n*=6; Welch's *t*-test). **(b-d)** WT, *Ephb2*$^{+/-}$ and *Ephb2*$^{-/-}$ mice were subjected to 60 min MCAO followed by 12 or 24 h of reperfusion as indicated. **(b)** Brain sections were stained with cresyl violet, and infarct and edema sizes were analyzed using ImageJ. Infarct volume is edema-corrected (single values (scatter blots) and mean±SD; *n*=6/5/6, 15/7/15; One-way ANOVA with Holm-Sidak's multiple comparisons test). **(c)** Neurological function was assessed using the Bederson neurological deficit score (single values and median; *n*=8/5/11, 17/7/15; Kruskal-Wallis test). **(d)** Motor coordination of mice

subjected to 60 min MCAO followed by 24 h of reperfusion was analyzed by using the Rotarod performance test (single values and mean±SD; *n*=5/7/4; Two-way ANOVA with Holm-Sidak's multiple comparisons test). * *p*<0.05.

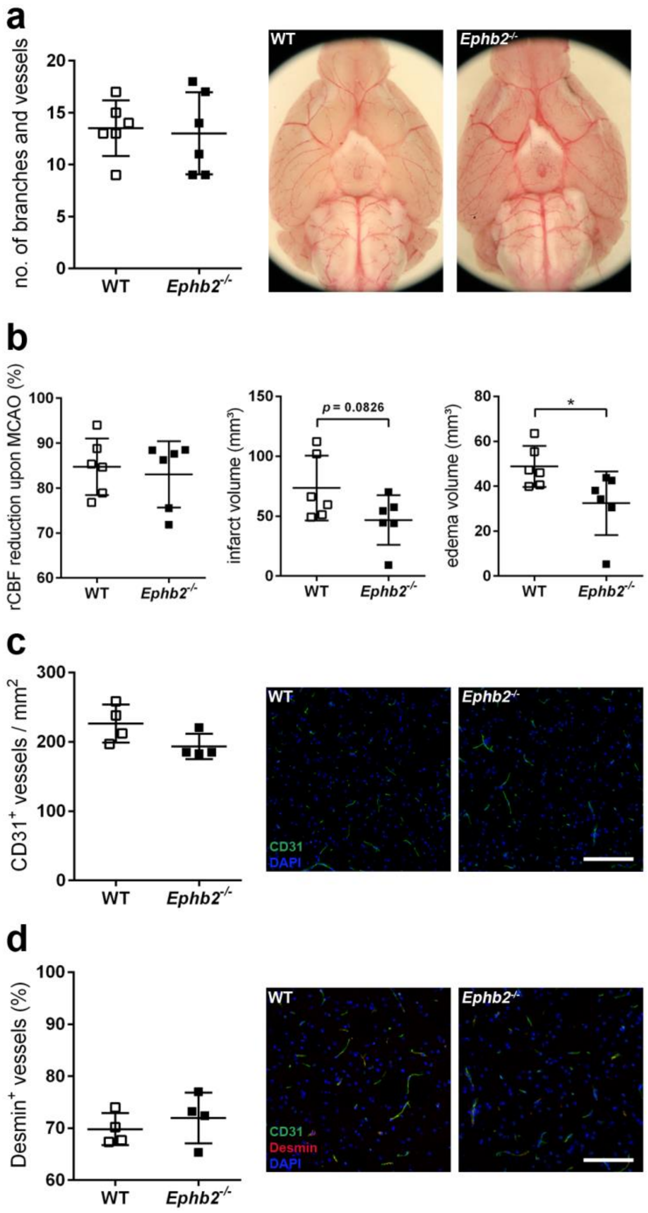

Fig. S3 EphB2 deficiency does not affect anatomy of the cerebral vascular system. **(a)** In naïve WT and *Ephb2*$^{-/-}$ mice the cerebral vasculature was stained through transcardial pigment particle perfusion. For each mouse the number of lenticulostriate arteries arising from the left or right proximal middle cerebral artery arterial branch was quantified (mean±SD; *n*=6; Student's *t*-test). **(b)** Brain perfusion deficit upon MCAO is not affected by the loss of EphB2. WT and *Ephb2*$^{-/-}$ mice were subjected to 60 min MCAO followed by 24 h of reperfusion. rCBF was measured just before and immediately after MCAO using LDF (single values and mean±SD; *n*=6/6; Mann-Whitney *U* rank-sum test). Brain sections were stained with cresyl

violet, and infarct and edema sizes were analyzed using ImageJ. Infarct volume is edema-corrected (single values and mean±SD; n=6/6; Student's t-test). * p<0.05. **(c, d)** Immunofluorescent detection of the marker proteins CD31 (endothelial cells) and desmin (pericytes) was used to determine **(c)** the density of cerebral microvessels, and **(d)** the pericyte-coverage of brain microvasculature (mean±SD; n=4; Student's t-test). Scale bar = 100 µm.

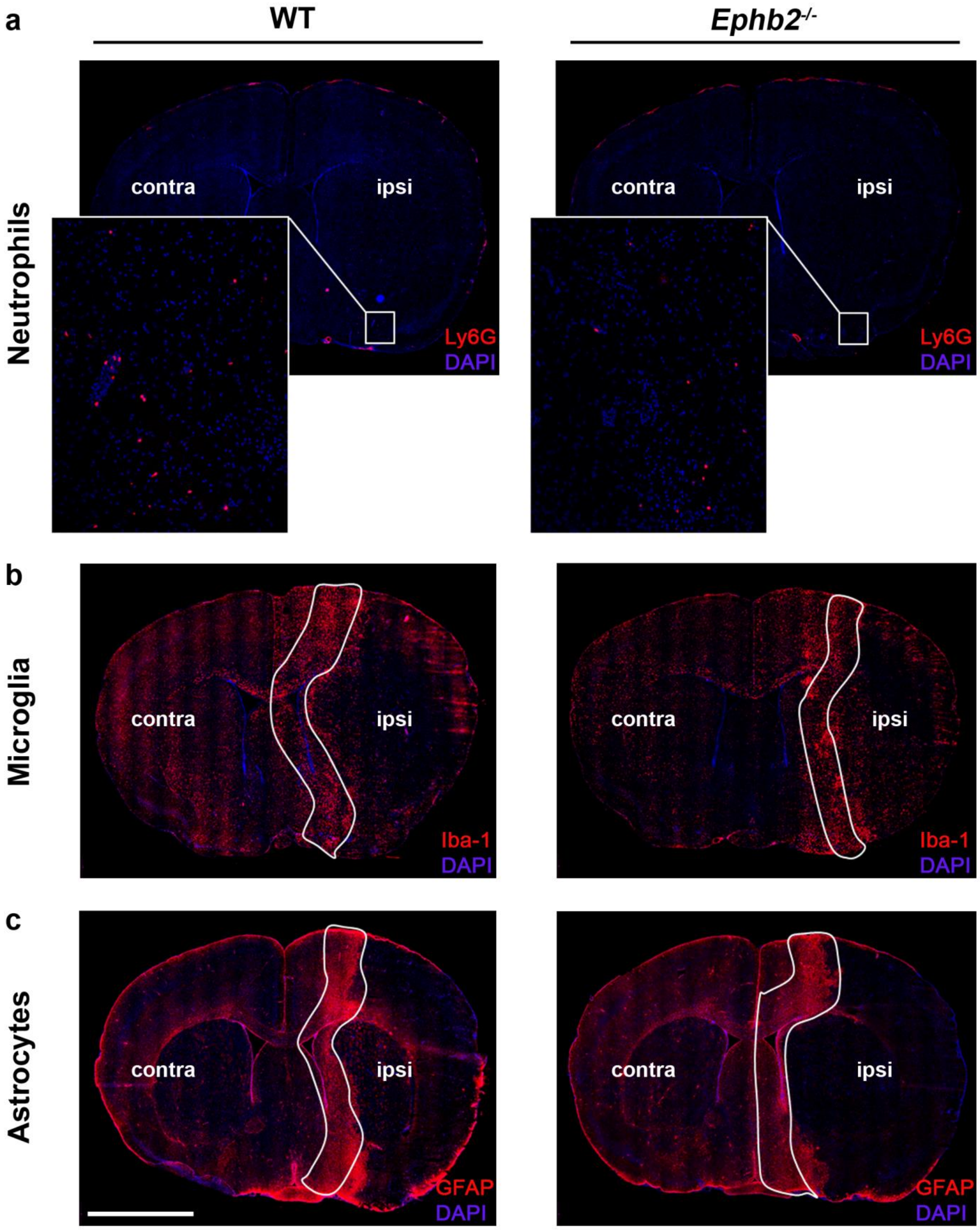

Fig. S4 Immunofluorescent detection of peripheral and resident immunocompetent cells in the CNS after acute stroke. WT and *Ephb2*$^{-/-}$ mice (n=4/group) were subjected to 60 min MCAO followed by **(a)** 24 h or **(b, c)** 72 h of reperfusion. Immunofluorescent detection of the marker proteins Ly6G, Iba-1 and GFAP in coronal brain sections was used to determine the number and spatial distribution of **(a)** neutrophils, **(b)** resident/activated microglia/macrophages and **(c)** astrocytes. In representative microphotographs the infarct border zones, defined as cresyl

violet-positive area next to the cresyl violet-negative infarct region, are completely encircled by white lines. Scale bar = 5 mm.

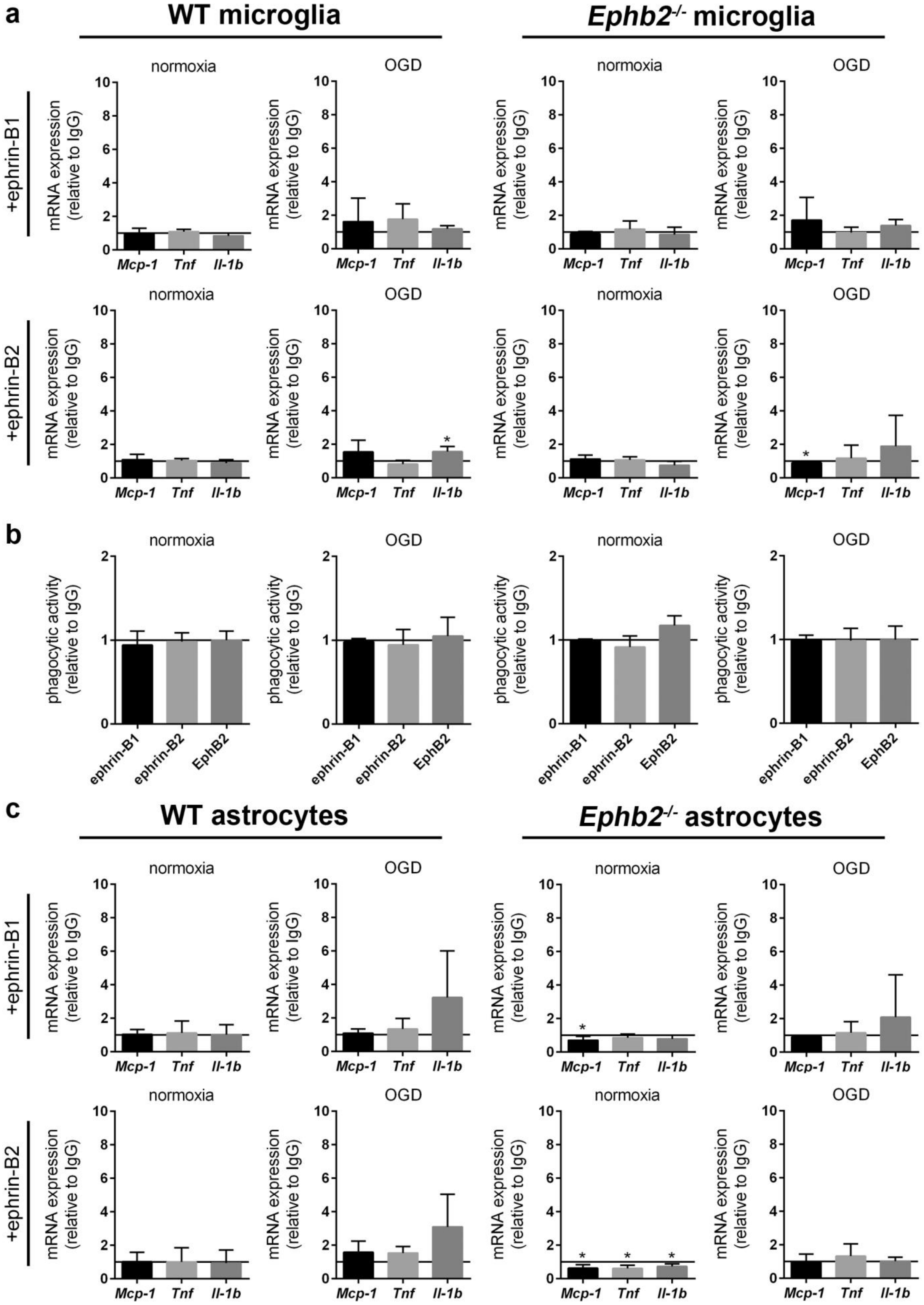

Fig. S5 EphB2/ephrin-B forward signaling does not induce pro-inflammatory activation of microglia and astrocytes. **(a, b)** Primary microglia isolated from brains of neonatal WT and *Ephb2*$^{-/-}$ mice were exposed to normoxic or OGD conditions for 6 h in the presence of either 10 nmol pre-clustered ephrin-B1/Fc, ephrin-B2/Fc, EphB2/Fc or anti-IgG Fc. **(a)** Gene expression was determined by quantitative real-time RT-PCR (mean±SD; *n*=5/3 (N), *n*=4/3 (OGD); Student's *t*-test). **(b)** Phagocytic activity was evaluated by quantifying the amount of incorporated fluorescent microspheres with fluorescent spectroscopy (mean±SD; *n*=3/3 (N), *n*=3/3 (OGD); Student's *t*-test). **(c)** Primary astrocytes derived from brains of new born WT and *Ephb2*$^{-/-}$ mice were exposed to normoxic or OGD conditions for 6 h in the presence of either 10 nmol pre-clustered ephrin-B1/Fc, ephrin-B2/Fc or anti-IgG Fc. Gene expression was analyzed by quantitative real-time RT-PCR (mean±SD; *n*=6/4 (N), *n*=3/4 (OGD); Student's *t*-test). * p<0.05.

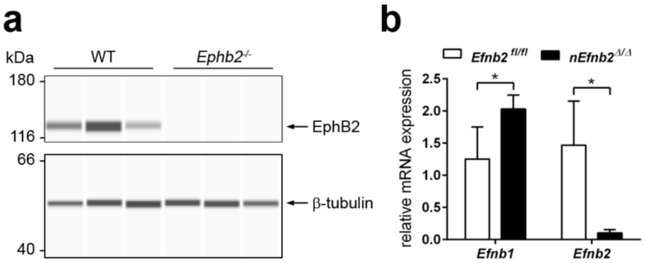

Fig. S6 Characterization of *Ephb2* and *Efnb2* gene ablation in primary astroglial cultures. **(a)** Capillary electrophoresis was used to determine the EphB2 protein amount in primary astrocytes isolated from brains of neonatal WT and *Ephb2*$^{-/-}$ mice (*n*=3/3). **(b)** Expression of *Efnb1* and *Efnb2* in primary astrocytes derived from brains of new born *Efnb2*$^{fl/fl}$ and *nEfnb2*$^{\Delta/\Delta}$ mice was evaluated by quantitative real-time RT-PCR (mean±SD; *n*=6/4; Student's *t*-test). * p<0.05.

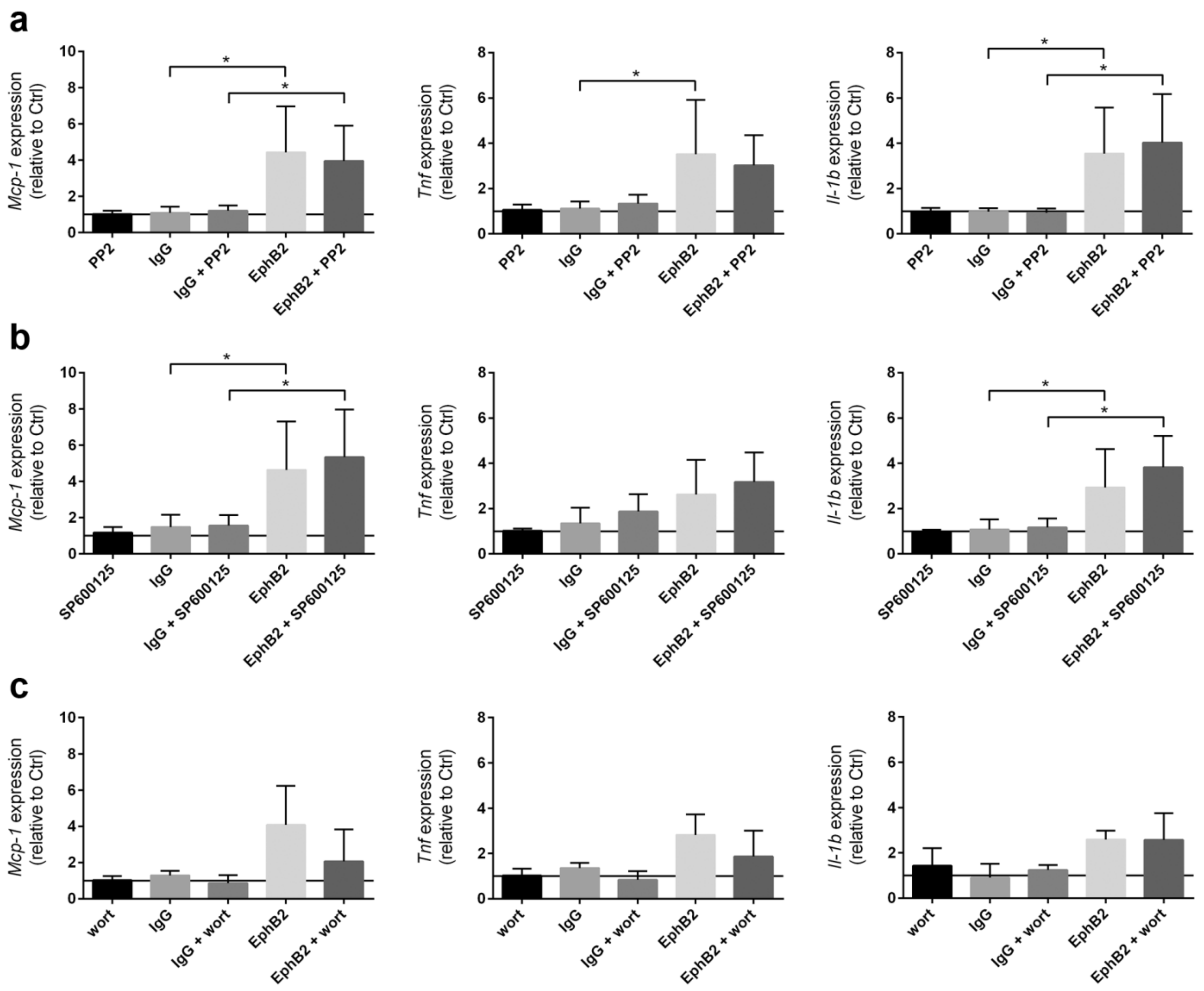

Fig. S7 Pro-inflammatory activation of astrocytes by EphB2 does not require Src-, JNK- or PI3K-dependent signaling pathways. Astrocytes isolated from brains of neonatal WT mice were treated with either **(a)** 10 µM PP2, **(b)** 20 µM SP600125 or **(c)** 10 nM wortmannin for 1 h prior to stimulation with pre-clustered EphB2/Fc or anti-IgG Fc for 6 h. Gene expression was analyzed by quantitative real-time RT-PCR (mean±SD; *n*=6 (PP2), *n*=5 (SP600125), *n*=3 (wortmannin); One-way ANOVA with Holm-Sidak's multiple comparisons test). * $p<0.05$.

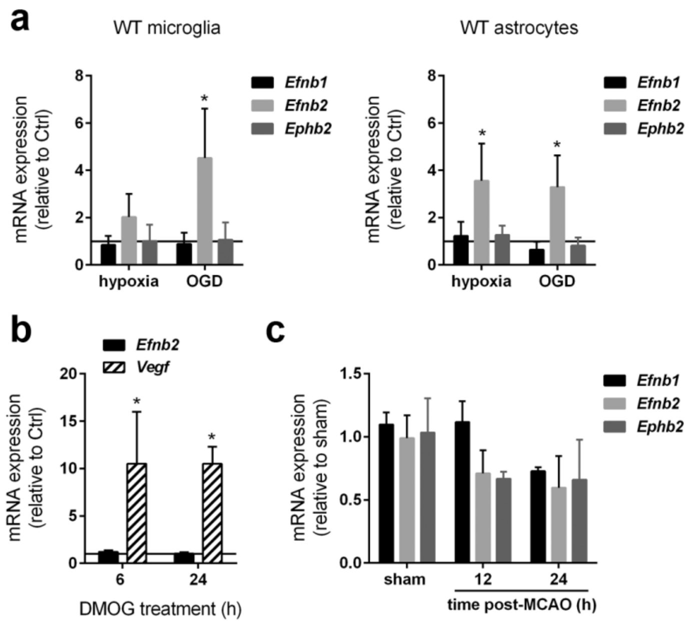

Fig. S8 Hypoxic or ischemic stress leads to HIF-independent up-regulation of ephrin-B2 in glial cells. **(a)** Microglia and astrocytes originating from brains of new born WT mice were exposed to either normoxic, hypoxic or OGD conditions for 6 h Gene expression was analyzed by quantitative real-time RT-PCR (mean±SD; *n*=6 (microglia), *n*=6 (astrocytes); One-way ANOVA with Holm-Sidak's multiple comparisons test). **(b)** Astrocytes were treated with 1 mM DMOG for 6 or 24 h. Cells treated with 0.1% DMSO for 24 h served as control (Ctrl). Gene expression was determined by quantitative real-time RT-PCR (mean±SD; *n*=4; One-way ANOVA with Holm-Sidak's multiple comparisons test). **(c)** WT mice underwent 60 min MCAO followed by 12 or 24 h of reperfusion or were subjected to sham surgery. RNA was extracted from ipsilesional brain tissue and corresponding tissue of sham operated mice. Gene expression was evaluated by quantitative real-time RT-PCR (mean±SD; *n*=3; One-way ANOVA with Holm-Sidak's multiple comparisons test). * $p<0.05$.

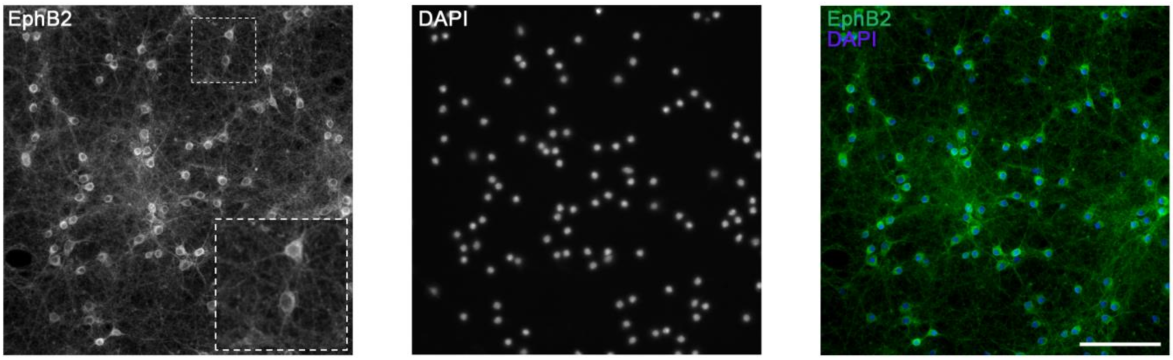

Fig. S9 Primary murine neurons are a source of EphB2 surface proteins. Immunofluorescent staining was used to detect EphB2 protein in primary murine neurons. Representative immunofluorescent staining images: EphB2 (green) and nuclei (blue). Scale bar = 100 μm.

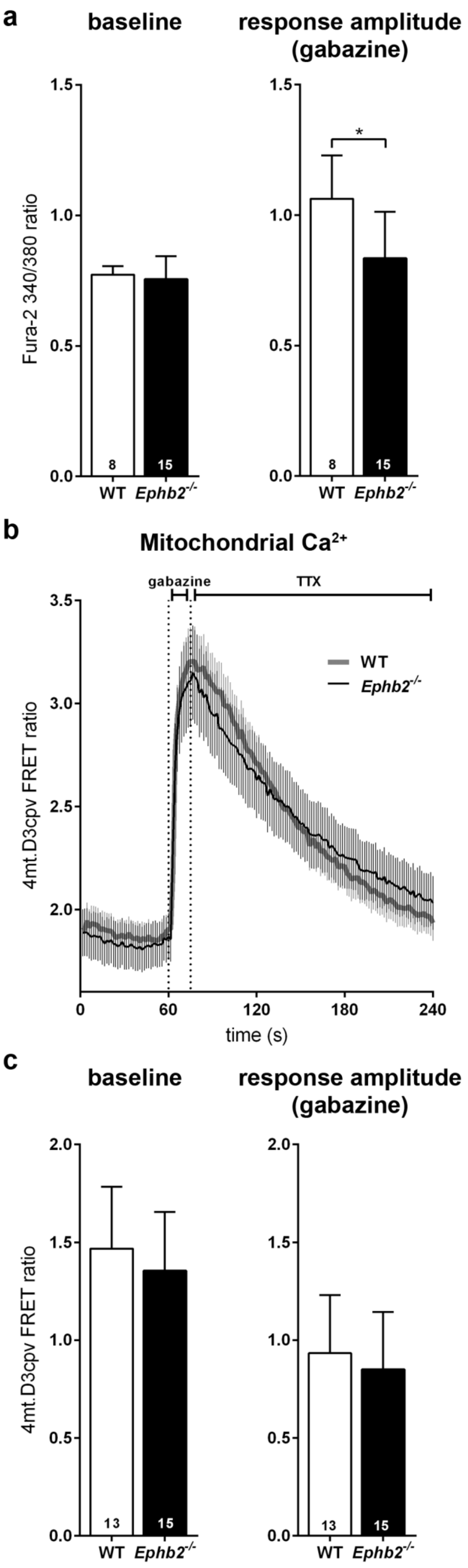
a
baseline
response amplitude (gabazine)
Fura-2 340/380 ratio
1.5
1.0
0.5
0.0
*
8
15
WT
Ephb2-/-
b
Mitochondrial Ca2+
gabazine
TTX
3.5
3.0
2.5
2.0
WT
Ephb2-/-
4mt.D3cpv FRET ratio
0
60
120
180
240
time (s)
c
baseline
response amplitude (gabazine)
4mt.D3cpv FRET ratio
2.0
1.5
1.0
0.5
0.0
13
15
WT
Ephb2-/-

Fig. S10 Cytoplasmic and mitochondrial Ca^{2+} levels during AP bursting. WT and *Ephb2*$^{-/-}$ forebrain neurons were obtained from P0 mice. Ca^{2+} imaging was performed in control conditions and during a single AP burst triggered by the GABAaR antagonist gabazine and halted by TTX. **(a)** Quantification of basal cytoplasmic Ca^{2+} levels and the amplitude of AP bursts probed with the ratiometric indicator Fura-2 (mean±SD; *n*=8/15 coverslips from 4 independent preparations; Student's *t*-test. **(b, c)** Mitochondrial Ca^{2+} imaging using the FRET-based indicator 4mt.D3cpv prior to and during a single gabazine-evoked AP burst. **(b)** Representative data from one coverslip each of WT and *Ephb2*$^{-/-}$ cells prior to and during stimulation with gabazine (mean±SEM). **(c)** Quantification of the baseline 4mt.D3cpv FRET ratio and the peak amplitude during the AP burst (mean±SD; *n*=13/15 coverslips from 4 independent preparations; Student's *t*-test). * $p<0.05$.
