# Supplementary material for: EphB2-dependent signaling promotes neuronal excitotoxicity and inflammation in the acute phase of ischemic stroke
Source: Acta Neuropathol Commun. 2019 Feb 5;7:15. doi: 10.1186/s40478-019-0669-7 (PMC6362601; doi:10.1186/s40478-019-0669-7)
Supplement: Supplementary file 2 — Table S1. List of primers used to genotype mice. Table S2. Overview of animals that met defined exclusion criteria. Table S3. Primary antibodies used for immunofluorescent staining. Table S4. List of primers used for quantitative real-time RT-PCR. Table S5. KEGG pathway-Based gene set enrichment analyses (GSEA). (PDF 271 kb) [file 40478_2019_669_MOESM2_ESM.pdf]

ADDITIONAL FILE 2

EphB2-dependent signaling promotes neuronal excitotoxicity and inflammation in the acute phase of ischemic stroke

Anne-Sophie Ernst [1,*], Laura-Inès Böhler [1,*], Anna M. Hagenston [2], Angelika Hoffmann [3], Sabine Heiland [4], Carsten Sticht [5], Martin Bendszus [3], Markus Hecker [1], Hilmar Bading [2], Hugo H. Marti [1], Thomas Korff [1,*], Reiner Kunze [1,*]

[1] Institute of Physiology and Pathophysiology, Department of Cardiovascular Physiology, Heidelberg University, Heidelberg, Germany

[2] Department of Neurobiology, Interdisciplinary Center for Neurosciences, Heidelberg University, Heidelberg, Germany

[3] Department of Neuroradiology, Heidelberg University Hospital, Heidelberg, Germany

[4] Division of Experimental Radiology, Department of Neuroradiology, Heidelberg University Hospital, Heidelberg, Germany

[5] Center of Medical Research, Medical Faculty Mannheim, Heidelberg University, Heidelberg, Germany

[*] equal contribution

Address for correspondence:

Hugo H. Marti

Heidelberg University

Institute of Physiology and Pathophysiology

Im Neuenheimer Feld 326

69120 Heidelberg, Germany

Phone 49-6221-544138

FAX 49-6221-548224

E-mail hugo.marti@physiologie.uni-heidelberg.de

Supplementary Tables

Table S1 List of primers used to genotype mice.

Allele	Primer name	Primer sequence (5´-3´)	Size (bp) of amplicon
Nestin:cre	Cre1	ACCAGGTTCGTTCACTCATGG	217
	Cre2	AGGCTAAGTGCCTTCTCTACAC	
Efnb2 floxed	B2Cas1	TGCTTGATTGAAACGAAGCCCGA	wt: 240
	B2Cs1	CTTCAGCAATATACACAGGATG	floxed: 320
Ephb2 null	EphB2-F1	AAA CCC TGA TGG ACT CTA CGA CAG C	wt: 600
	Neo3	GTC AGT TTC ATA GCC TGA AGA ACG	null: 300
	N1B	GGG TAC ATC TCA GTG GTA GAA TG	

Table S2 Overview of animals that met defined exclusion criteria.

Animals that met the following criteria were excluded from end-point analyses: (1) death during surgery due to procedural or anesthetic problems, (2) death before sampling, (3) intracerebral hemorrhage, (4) structural brain abnormalities (e.g. hydrocephalus), and (5) no infarction.

mouse line	number of mice met exclusion criteria (% of total animals)					
	(1)	(2)	(3)	(4)	(5)	total
WT	5	1	1	1	2	10 / 99 (10%)
Ephb2$^{+/-}$	-	-	-	1	-	1 / 14 (7%)
Ephb2$^{-/-}$	-	-	-	2	2	4 / 86 (5%)
Efnb2$^{f/f}$	-	-	-	-	-	0 / 6 (0%)
nEfnb2$^{\Delta/\Delta}$	1	-	-	-	-	1 / 6 (17%)

Table S3 Primary antibodies used for immunofluorescent staining.

**Antibody**	**Supplier, Catalogue number**	**Dilution**	**Fixation**	**Blocking**
CD31	BD Biosciences, #557355	1:100	zinc-based	goat serum
desmin	Dianova, # DLN-13732	1:400	zinc-based	BSA/casein
EphB2	R&D Systems, #AF467	1:2000	zinc-based	BSA/casein
EphB4	R&D Systems, #AF446	1:1000	zinc-based	BSA/casein
ephrin-B1	R&D Systems, #AF473	1:4000	zinc-based	BSA/casein
ephrin-B2	R&D Systems, #AF496	1:500	zinc-based	BSA/casein
GFAP	Agilent, #Z0334	1:1000	zinc-based	BSA/casein
Iba-1	FUJIFILM Wako, #019-19741	1:500	1% PFA	BSA/casein
Ly6G	BD Biosciences, #551459	1:250	1% PFA	Sea block
NeuN	Bio-Techne, #NBP1-92693	1:500	2% PFA	BSA/casein
NF-kB	BioLegend, #622602	1:200	3% PFA	Sea block
ZO-1	Thermo Fisher Scientific, #61-7300	1:100	zinc-based	goat serum

Table S4 List of primers used for quantitative real-time RT-PCR.

Gene	Forward primer sequence (5´-3´)	Reverse primer sequence (5´-3´)
Cox2	AGAAGGAAATGGCTGCAGAA	GCTCGGCTTCCAGTATTGAG
Efnb1	ATCGCAAGCATACACAGCAG	TGGGGGCAGTAGTTGTTCTC
Efnb2	AGGAATCACGGTCCAACAAG	GTCTCCTGCGGTACTTGAGC
Efnb2 exon2	TCAACTGTGCCAGACCAGAC	TAGACCCCAGAGGTTAGGGC
Ephb2	ACTATGGCGGCTGTATGTCC	GCACATCCACTTCTTCAGCA
Il-1b	ATAACCTGCTGGTGTGTGACG	GGTGGAGAGCTTTCAGCTCAT
Il-6	AGTTGCCTTCTTGGGACTGA	TCCACGATTTCCCAGAGAAC
Mcp-1	CCCAATGAGTAGGCTGGAGA	TCTGGACCCATTCCTTCTTG
Rps12	GAAGCTGCCAAAGCCTTAGA	AACTGCAACCAACCACCTTC
Tnf	CGTCAGCCGATTTGCTATCT	CGGACTCCGCAAAGTCTAAG
Vegfa	GTACCTCCATGCCAAGT	ACTCCAGGGCTTCATCGTTA

Cox2, Cyclooxygenase-2; *Efnb1/2*, ephrin-B1/2, *Ephb2*, EphB2; *Il-1b*, interleukin-1 beta; *Il-6*, interleukin-6; *Mcp-1*, monocyte chemoattractant protein-1; *Rps12*, ribosomal protein S12; *Tnf*, tumor necrosis factor alpha; *Vegf*, vascular endothelial growth factor

Table S5 KEGG pathway-Based gene set enrichment analyses (GSEA).

Ephb2$^{-/-}$ and WT mice (*n* = 3) were subjected to 60 min MCAO followed by 48 hours reperfusion. Total RNA was extracted from ipsilesional brain tissue and processed for DNA microarray analysis (GeneChip Mouse Gene 2.0 ST; Affymetrix, Santa Clara, CA, USA). The table lists certain significantly UP and DOWN-regulated gene sets comparing *Ephb2*$^{-/-}$ versus WT matching the following criteria for selection: ≥1.5- or ≤1.5-fold regulation (log2-fold), p < 0.05 (ANOVA), FDR < 0.1. NES: normalized enrichment score; FDR: false discovery rate.

	**PATHWAY NAME**	**NES**	**NOM p-val**	**FDR q-val**
cell death	FoxO signaling pathway	-1.80	<0.001	0.004
	Proteasome	-1.98	<0.001	0.001
	Apoptosis	-2.00	<0.001	0.001
	p53 signaling pathway	-2.13	<0.001	<0.001
inflammation and immune cell signaling	Platelet activation	-1.50	0.003	0.048
	NOD-like receptor signaling pathway	-1.55	0.011	0.031
	Chemokine signaling pathway	-1.76	<0.001	0.006
	Fc gamma R-mediated phagocytosis	-1.79	<0.001	0.005
	Toll-like receptor signaling pathway	-1.97	<0.001	0.001
	Antigen processing and presentation	-2.00	<0.001	0.001
	B cell receptor signaling pathway	-2.07	<0.001	<0.001
	Leukocyte transendothelial migration	-2.15	<0.001	<0.001
	Complement and coagulation cascades	-2.16	<0.001	<0.001
	TNF signaling pathway	-2.17	<0.001	<0.001
	NF-kappa B signaling pathway	-2.20	<0.001	<0.001
	Cytokine-cytokine receptor interaction	-2.23	<0.001	<0.001
synaptic function	Glutamatergic synapse	2.08	<0.001	0.001
	Retrograde endocannabinoid signaling	1.97	<0.001	0.001
	GABAergic synapse	1.87	<0.001	0.005
	Axon guidance	1.83	<0.001	0.007
	Calcium signaling pathway	1.77	<0.001	0.011
	Dopaminergic synapse	1.51	0.005	0.082
