# Supplementary material for: EphB2-dependent signaling promotes neuronal excitotoxicity and inflammation in the acute phase of ischemic stroke
Source: Acta Neuropathol Commun. 2019 Feb 5;7:15. doi: 10.1186/s40478-019-0669-7 (PMC6362601; doi:10.1186/s40478-019-0669-7)
Supplement: Supplementary file 3 — Supplementary Methods. (PDF 204 kb) [file 40478_2019_669_MOESM3_ESM.pdf]

ADDITIONAL FILE 3

EphB2-dependent signaling promotes neuronal excitotoxicity and inflammation in the acute phase of ischemic stroke

Anne-Sophie Ernst [1,*], Laura-Inès Böhler [1,*], Anna M. Hagenston [2], Angelika Hoffmann [3], Sabine Heiland [4], Carsten Sticht [5], Martin Bendszus [3], Markus Hecker [1], Hilmar Bading [2], Hugo H. Marti [1], Thomas Korff [1,*], Reiner Kunze [1,*]

[1] Institute of Physiology and Pathophysiology, Department of Cardiovascular Physiology, Heidelberg University, Heidelberg, Germany

[2] Department of Neurobiology, Interdisciplinary Center for Neurosciences, Heidelberg University, Heidelberg, Germany

[3] Department of Neuroradiology, Heidelberg University Hospital, Heidelberg, Germany

[4] Division of Experimental Radiology, Department of Neuroradiology, Heidelberg University Hospital, Heidelberg, Germany

[5] Center of Medical Research, Medical Faculty Mannheim, Heidelberg University, Heidelberg, Germany

[*] equal contribution

Address for correspondence:
Hugo H. Marti
Heidelberg University
Institute of Physiology and Pathophysiology
Im Neuenheimer Feld 326
69120 Heidelberg, Germany
Phone 49-6221-544138
FAX 49-6221-548224
E-mail hugo.marti@physiologie.uni-heidelberg.de

Supplementary Methods

Glial cell cultures

Primary astroglial and microglial cultures from neonatal mice (P0-P2) were prepared and maintained as described previously [6].

Pharmacological treatments. Cells were treated for 6 hours with either ephrin-B1/IgG, ephrin-B2/IgG or EphB2/IgG clusters, prepared by incubation of equimolar amounts of rmephrin-B1/Fc chimera protein (R&D Systems, Wiesbaden, Germany, #473-EB-200; 10 µM), rmephrin-B2/Fc (R&D Systems, #496-EB-200; 10 µM), rmEphB2/Fc (R&D Systems, #467-B2-200; 10 µM) and anti-human IgG Fc (R&D Systems, #MAB110-500; 10 µM), respectively, in medium for 1 hour at room temperature. Cells treated with 10 µM anti-human IgG Fc were used as control. For certain experiments, astrocytes were treated with either Bay 11-7082 (Cayman Chemical, Ann Arbor, MI, USA, #10010266; 10 µM), PP2 (Abcam, Berlin, Germany, #ab120308; 10 µM), SP600125 (Abcam, #ab120065; 20 µM), Wortmannin (Merck Millipore, Darmstadt, Germany, #681675; 10 nM), PD98059 (Merck Millipore, #513000; 20 µM), or SB203580 (Merck Millipore, #559395; 10 µM) for 1 hour prior to stimulation with anti-IgG Fc or clustered EphB2/IgG.

Oxygen-glucose deprivation (OGD). Cells were cultured in deoxygenated glucose-free Dulbecco's Modified Eagle Medium (Thermo Fisher Scientific, Darmstadt, Germany, #11966025) supplemented with 1 % FBS in an Invivo2 Plus hypoxia workstation (Ruskinn, Leeds, UK) flooded with humidified gas mixture consisting of 5 % CO_2 and ~94 % N_2 (1 % O_2) at 37 °C for 6 hours in the presence of either 10 nmol pre-clustered EphB2/Fc, ephrin-B1/Fc, ephrin-B2/Fc or anti-IgG Fc. Gene expression and microglial phagocytosis were determined directly after OGD.

Analysis of microglial phagocytosis. Microglial cells were treated with anti-IgG Fc, ephrin-B1/IgG, ephrin-B2/IgG or EphB2/IgG under either normoxic or OGD conditions as described

above. Fluorochrome-labeled latex microspheres (Thermo Fisher Scientific, #F8815; 1:250) were added 30 minutes before end of ephrin-B/EphB2 stimulation and incubated for 30 minutes. Isolation and quantification of phagocytosed fluorescent microspheres were performed according to manufacturer's instructions.

Neuronal Cell Culture

Cortical neurons from newborn mice were prepared and maintained as described previously [3, 7]. Experiments were performed after a culturing period of 10-11 days *in vitro* (DIV) when neurons express functional glutamate receptors and have developed an extensive network of synaptic contacts. At DIV 8 growth medium was replaced with defined medium consisting of a mixture of a buffered saline solution (10 mM HEPES, pH 7.4, 114 mM NaCl, 26.1 mM $NaHCO_3$, 5.3 mM KCl, 1 mM $MgCl_2$, 2 mM $CaCl_2$, 30 mM glucose, 1 mM glycine, 0.5 mM $C_3H_3NaO_3$, and 0.001% phenol red) and phosphate-free Eagle's minimum essential medium (9:1 v/v) supplemented with insulin (7.5 μg/ml), transferrin (7.5 μg/ml), and sodium selenite (7.5 ng/ml) (ITS supplement, Sigma-Aldrich, Steinheim, Germany). The following drugs were used in this study: FCCP (Sigma-Aldrich, #C2920; 5 μM), gabazine (SR 99531 hydrobromide; Biotrend, Cologne, Germany, #BN 0507; 5 μM), NBQX (Hello Bio, Bristol, UK, #HB0443; 2 μM), nifedipine (Biotrend, #A10643; 7 μM), NMDA (Biotrend, #BN0385; 5-30 μM), TTX (Biotrend, #BN0518; 1 μM) and verapamil (Bio-Techne, Wiesbaden, Germany, #0654; 30 μM).

Recombinant adeno-associated viruses (rAAVs). Viral particles were produced and purified as described previously [7]. To drive expression of the mitochondrially targeted, FRET-based Ca^{2+} indicator 4mtD3cpv, we used a viral vector containing a CKIIα promoter. Cultured neurons were infected with ~10^{11} rAAV particles/ml on DIV 3-4 and analyzed on DIV 10-11.

Imaging. All imaging experiments were performed at room temperature in a HEPES-buffered saline solution (HBS) containing 140 mM NaCl, 2.5 mM KCl, 1.0 mM $MgCl_2$, 2.0 mM

$CaCl_2$, 10 mM HEPES, 1.0 mM glycine, 35.6 mM D-glucose, and 0.5 mM $C_3H_3NaO_3$. Changes in mitochondrial Ca^{2+} levels and membrane potential were analyzed as described [2, 4, 5] using the FRET-based, mitochondrially targeted Ca^{2+} indicator, 4mtD3cpv, and the small molecule dye, Rh123, respectively. Changes in cytoplasmic calcium levels were analyzed using the small molecule dye, Fura-2. Fluorescence images were acquired at 0.667-2.0 Hz using a cooled CCD camera (iXon, Andor, Belfast, UK) through a 20x water-immersion objective (XLMPlanFluor, Olympus, Hamburg, Germany) on an upright microscope (BX51W1, Olympus). Fluorescence excitation (4mtD3cpv: CFP 430±12 nm, YFP 500±10 nm; Fura-2: 340±10 nm and 380±10 nm; Rh123: 470±20 nm; AHF Analysentechnik, Tübingen, Germany) was provided by a xenon arc lamp in combination with an excitation filter wheel (cell^R, Olympus). Fura-2 and Rh123 fluorescence were filtered using 510±20 nm and 525±25 nm emission filters, respectively (AHF Analysentechnik). For 4mtD3cpv imaging, CFP (470±12 nm) and YFP (535±15 nm) emission wavelengths were separated and filtered using a DualView beam splitter (AHF Analysentechnik and MAG Biosystems, Tucson, AZ, USA). Data were collected using cell^R software (Olympus) and analyzed using Fiji (RRID:SCR_02283) and IgorPro (WaveMetrics, Portland, OR, USA, RRID:000325). Mitochondrial Ca^{2+} concentration changes in regions of interest drawn around individual neurons were quantified using the crosstalk- and bleaching-corrected 4mtD3cpv FRET ratio [4]. For mitochondrial membrane potential (Ψm) imaging, cells grown on coverslips were loaded with Rh123 (Thermo Fisher Scientific, #R302; 4.3 μM) in HBS for 30 minutes at room temperature followed by extensive washing with HBS. Due to its positive charge, Rh123 accumulates under basal conditions within the mitochondrial matrix, where its high concentration leads to quenching. Mitochondrial membrane depolarization induces leakage of Rh123 from the mitochondria into the cytoplasm, where its fluorescence is dequenched resulting in an increase in fluorescence intensity [1]. Maximum Rh123 signal was obtained by exposing the recorded cells to the mitochondrial uncoupler FCCP (Sigma-Aldrich, #C2920; 5

μM). Rh123 fluorescence levels were measured in the nucleus to avoid possible contamination by fluorescence signals emerging from mitochondria and quantified on a cell-by-cell basis as a % of the FCCP-induced level for that cell. For cytoplasmic Ca^{2+} imaging, cells grown on coverslips were loaded with Fura-2-AM (Thermo Fisher Scientific, #F1221; 1 μM; stock solution, 1 mM in 20% Pluronic F-127 (Sigma-Aldrich, #P2443) in DMSO) in HBS for 30 minutes at 37°C followed by extensive washing with HBS at 37°C. Cytoplasmic Ca^{2+} concentration changes in regions of interest drawn around individual neurons were quantified using the ratio of emission intensities obtained by sequential excitation with 340±10 nm and 380±10 nm light (340/380 ratio).

Immunofluorescence analysis

Staining. Coronal brain cryosections (10 μm in thickness; +0.62 to -0.62 mm relative to Bregma) and cell monolayer were fixed, permeabilized with 0.5% saponin in PBS for 10 minutes (only CD31, NeuN, NF-κB, ZO-1) and incubated with blocking buffer as indicated in Table S3 (Additional file 2). Fixation was performed either with a zinc-based fixative (100 mM Tris (pH 7.4), 3 $Ca(C_2H_3O_2)_2$, 25 mM $Zn(C_2H_3O_2)_2$ and 35 mM $ZnCl_2$) for 30 minutes or with 1% PFA in PBS for 20 minutes or with 2-3% PFA in PBS for 15 minutes. Blocking was performed either with 10% goat serum in PBS-T or 0.1% BSA, 0.25% casein, 15 mM NaN_3 in 50 mM Tris (pH 7.6) or 10% Sea block (Thermo Fisher Scientific, #37527) for 30 minutes. Tissue sections or cells were incubated with primary antibodies overnight at 4°C as described in Table S3 (Additional file 2). Then, sections or cells were incubated with appropriate secondary antibodies for 1 hour and counterstained with DAPI (1 μg/mL in PBS, 3 minutes; Thermo Fisher Scientific, #D1306). All antibodies were diluted in LowCross-Buffer (Candor Bioscience, Wangen, Germany #100125). Subsequently, stained sections or cells were embedded in Mowiol mounting medium (Polysciences, Hirschberg an der Bergstrasse, Germany, #17951) and stored at 4°C until imaging.

For immunofluorescent staining of EphB/ephrin-B proteins, endogenous peroxidases were blocked with 0.3% H_2O_2 in methanol for 10 minutes after fixation. Prior to blocking, sections or cells were washed with H_2O. After incubation with the primary antibody overnight, sections or cells were washed with TBS-T and incubated for 1 hour with a secondary anti-goat IgG antibody (R&D Systems, #R-401-C-ABS; 1:500). Sections were washed with TBS-T and incubated for 30 minutes with DAKO-EnVision rabbit-HRP (Agilent, Hamburg, Germany, K4002). Upon washing with TBS-T, sections were treated with a Tyramide Signal Amplification reagent (PerkinElmer, Rodgau, Germany; #NEL745001KT; 1:100) for 1 minute, prior to washing with TBS-T and subsequent counterstaining with DAPI as described above.

Imaging and analysis. Fluorescence staining in tissue sections and cell monolayer was recorded using a Zeiss Axiovert 200M microscope (Carl Zeiss Microscopy, Göttingen, Germany) with a Hamamatsu ORCA flash 4.0 camera (Hamamatsu Photonics, Herrsching am Ammersee, Germany) and an Olympus IX51 microscope with high-resolution camera (Olympus), respectively.

To quantify the density of brain vessels, areas with CD31-specific fluorescence intensity were assigned as vessels. Areas, which were associated with desmin-specific fluorescence intensity was defined as an area of a vessel covered by a pericyte. The number of vessels and pericyte-coverage was quantified using the image analysis software ImageJ (National Institutes of Health, Bethesda, MD, USA). ImageJ macros were designed by Dr. Holger Lorenz (Center for Molecular Biology of Heidelberg University, Heidelberg, Germany).

To analyze purity of neuronal, astrocytic and microglial cell cultures, 10 evenly distributed fields of view of each preparation were imaged. Nuclei were identified by DAPI staining. Cells showing NeuN-, GFAP- or Iba-1-specific fluorescent signal were identified as neurons, astrocytes or microglia, respectively. Shrunken nuclei showing high fluorescent intensity of DAPI staining were considered as dead and excluded from quantification. Automatic analysis

was performed utilizing TissueQuest 4.0 software (TissueGnostics, Vienna, Austria) or ImageJ.

Supplementary References

1. Baracca A, Sgarbi G, Solaini G, Lenaz G (2003) Rhodamine 123 as a probe of mitochondrial membrane potential: evaluation of proton flux through F(0) during ATP synthesis. Biochim Biophys Acta 1606(1-3):137-146

2. Hardingham GE, Fukunaga Y, Bading H (2002) Extrasynaptic NMDARs oppose synaptic NMDARs by triggering CREB shut-off and cell death pathways. Nat Neurosci 5(5):405-414

3. Lau D, Bengtson CP, Buchthal B, Bading H (2015) BDNF Reduces Toxic Extrasynaptic NMDA Receptor Signaling via Synaptic NMDA Receptors and Nuclear-Calcium-Induced Transcription of inhba/Activin A. Cell Rep 12(8):1353-1366

4. Palmer AE, Tsien RY (2006) Measuring calcium signaling using genetically targetable fluorescent indicators. Nat Protoc 1(3):1057-1065

5. Qiu J, Tan YW, Hagenston AM, Martel MA, Kneisel N, Skehel PA, Wyllie DJ, Bading H, Hardingham GE (2013) Mitochondrial calcium uniporter Mcu controls excitotoxicity and is transcriptionally repressed by neuroprotective nuclear calcium signals. Nat Commun 4:2034

6. Regen T, van Rossum D, Scheffel J, Kastriti ME, Revelo NH, Prinz M, Bruck W, Hanisch UK (2011) CD14 and TRIF govern distinct responsiveness and responses in

mouse microglial TLR4 challenges by structural variants of LPS. Brain Behav Immun 25(5):957-970

7. Zhang SJ, Steijaert MN, Lau D, Schutz G, Delucinge-Vivier C, Descombes P, Bading H (2007) Decoding NMDA receptor signaling: identification of genomic programs specifying neuronal survival and death. Neuron 53(4):549-562
